# The RORɣ/SREBP2 pathway is a master regulator of cholesterol metabolism and serves as potential therapeutic target in *t(4;11)* leukemia

**DOI:** 10.1038/s41388-023-02903-3

**Published:** 2023-11-29

**Authors:** Estelle Erkner, Thomas Hentrich, Rebekka Schairer, Rahel Fitzel, Kathy-Ann Secker-Grob, Johan Jeong, Hildegard Keppeler, Fulya Korkmaz, Julia M. Schulze-Hentrich, Claudia Lengerke, Dominik Schneidawind, Corina Schneidawind

**Affiliations:** 1 Department of Hematology, Oncology, Clinical Immunology and Rheumatology, University Hospital Tuebingen, Tuebingen, Germany; 2 Department of Genetics/Epigenetics, Faculty NT, Saarland University, Saarbruecken, Germany; 3 Process Cell Sciences, Merck & Co., Inc., Kenilworth, NJ USA; 4 Department of Medical Oncology and Hematology, University Hospital Zurich, Zurich, Switzerland

**Keywords:** Leukaemia, Cancer metabolism

## Abstract

Dysregulated cholesterol homeostasis promotes tumorigenesis and progression. Therefore, metabolic reprogramming constitutes a new hallmark of cancer. However, until today, only few therapeutic approaches exist to target this pathway due to the often-observed negative feedback induced by agents like statins leading to controversially increased cholesterol synthesis upon inhibition. Sterol regulatory element-binding proteins (SREBPs) are key transcription factors regulating the synthesis of cholesterol and fatty acids. Since SREBP2 is difficult to target, we performed pharmacological inhibition of retinoic acid receptor (RAR)-related orphan receptor gamma (RORγ), which acts upstream of SREBP2 and serves as master regulator of the cholesterol metabolism. This resulted in an inactivated cholesterol-related gene program with significant downregulation of cholesterol biosynthesis. Strikingly, these effects were more pronounced than the effects of fatostatin, a direct SREBP2 inhibitor. Upon RORγ inhibition, RNA sequencing showed strongly increased cholesterol efflux genes leading to leukemic cell death and cell cycle changes in a dose- and time-dependent manner. Combinatorial treatment of *t(4;11)* cells with the RORγ inhibitor showed additive effects with cytarabine and even strong anti-leukemia synergism with atorvastatin by circumventing the statin-induced feedback. Our results suggest a novel therapeutic strategy to inhibit tumor-specific cholesterol metabolism for the treatment of *t(4;11)* leukemia.

## Introduction

*KMT2A*-rearranged leukemia (*KMT2A*r, former *MLL*r) is a chromosomal translocation resulting in the development of chimeric fusion genes and proteins with oncogenic potential. Translocations involving the *KMT2A* gene occur in nearly 10% of acute leukemias and are observed in both acute myeloid leukemia (AML) and acute lymphoblastic leukemia (ALL) [1, 2]. The *KMT2A* gene has been shown to fuse with more than 80 distinct partner genes, of which one of the most frequent is *AFF1* (former *AF4*), resulting in a *t(4;11)* leukemia [3]. Especially infants harboring a *t(4;11)* leukemia are considered as high-risk patients with poor prognosis [3–5]. To overcome the limitations of culturing primary *t(4;11)* leukemia cells and commercial cell lines showing other mutations [6, 7], we recently established a CRISPR/Cas9 *t(4;11)* leukemia model [8–10]. Our in vitro model was successfully established as a platform for the identification and testing of potential therapeutic targets providing a rationale for possible therapies of *KMT2A*r leukemias [9].

The alteration of cellular metabolic pathways is considered as a hallmark of cancer [11]. Cholesterol is a hydrophobic sterol molecule produced by every nucleated cell and serves as the precursor of downstream metabolites such as bile acids, steroid hormones and cholecalciferol. In addition to its function as an important component of cellular membranes, it serves as key modulator of transmembrane signaling [12, 13]. It has been reported that cholesterol metabolism promotes cell growth, inhibits apoptosis [14, 15] and contributes to drug resistance of leukemic cells [16, 17]. Even though there are discrepant results in intracellular cholesterol levels [18, 19], it is widely accepted that cholesterol biosynthesis is enhanced in leukemia and the uptake rate for cholesterol is more rapid [16, 20].

Cholesterol homeostasis is under tight regulation at the transcriptional and post-transcriptional level. Sterol regulatory element-binding protein 2 (SREBP2, encoded by the *SREBF2* gene) is known to be an important transcription factor and key regulator of sterol metabolism. In the presence of sterols, the inactive precursor form of SREBP2 is bound to SREBP cleavage-activating protein (SCAP), which in turn is bound to the insulin induced gene 1 protein (INSIG) in the membrane of the endoplasmic reticulum. At low intrinsic sterol concentrations, SCAP detaches from INSIG and transports SREBP2 to the Golgi apparatus, where it is proteolytically cleaved. The N-terminal active form of SREBP2 enters the nucleus and mediates the transcription of important sterol regulatory elements (SRE)-containing genes such as *HMGCR*, *LDLR*, *SQLE* and *ABCA1*. These cholesterol-related target genes are translated into key proteins involved in the biosynthesis of mevalonate-derived metabolites and in cholesterol uptake, storage and efflux [21, 22]. Hereby, most of the mevalonate in hepatocytes is converted to cholesterol in hepatocytes in several steps, with HMGCR being the rate-limiting enzyme. In terms of a negative feedback loop, subsequent synthesis products may in turn reduce the extent of SREBP2-mediated cholesterol synthesis and uptake, thus ensuring that the metabolic pathway remains in balance [22].

Statins are widely used to lower serum cholesterol levels by inhibiting HMGCR activity. The resulting lower LDL cholesterol content leads compensatorily to an increased synthesis of LDL receptors in hepatocytes with the consequence that more LDL cholesterol is taken up into the cells and serum levels decrease accordingly. Thus, in addition to the direct inhibition of cholesterol biosynthesis, there is also an indirect transfer of cholesterol from the blood into the cells [23]. In addition, there is a growing body of preclinical and epidemiological evidence suggesting that mevalonate pathway inhibitors (e.g., statins) can be used as anticancer drugs and preliminary clinical investigations showed their antileukemic potential [24–26]. However, these studies were limited by a small cohort and only favorable risk AML patients [27, 28]. In addition, clinical trials using statins for solid cancer therapy failed due to an elevated cholesterol metabolism as negative feedback loop in response to statin therapy [29, 30]. Similarly, studies with multiple myeloma cell lines showed a differential response in terms of sensitivity and insensitivity to lovastatin-induced apoptosis. The missing response in insensitive cells is explained by a statin-induced inhibition of HMGCR triggering a robust homeostatic feedback response including the activation of SREBP2. Here, this naturally occurring negative feedback response leads to an upregulation of the mevalonate pathway and allows statins to work as cholesterol-lowering agents. Importantly, statin-sensitive cells did not exhibit the expected feedback of mevalonate metabolism [31]. In contrast, inhibition of retinoic acid receptor (RAR)-related orphan receptor gamma (RORγ) abolished the entire cholesterol biosynthesis program, circumventing reactivation of metabolism and the feedback loop, as previously shown in studies with triple-negative breast cancer (TNBC) and prostate cancer cells. They have demonstrated that RORγ is an important driver of tumor growth, which could be successfully diminished by newly established RORγ antagonists [32, 33]. Importantly, RORγ has two isoforms and while RORγt (isoform 2) is highly expressed in sub-populations of immune cells and plays a critical role in the differentiation of Th17 cells, RORγ (isoform 1) has been shown to play an important role in controlling metabolic pathways [33]. Furthermore, RORγ has been identified as novel master regulator of cholesterol metabolism mimicking the transcriptional effect of SREBP2 and suppressing cholesterol efflux by ATP-binding cassette (ABC)-transporters such as ABCA1 and ABCG1 [33–35]. Especially the survival of TNBC cells has been shown to be strongly dependent on the function of RORγ in cholesterol homeostasis [34].

In this study, we focused on the SREBP2-mediated sterol metabolism in *t(4;11)* leukemia. Therefore, we influenced cholesterol homeostasis at different transcriptional levels and evaluated the respective antileukemic effects. We could show that the RORγ antagonist, named XY018, was the most promising agent compared to other cholesterol-modulating drugs. Pharmacological inhibition of RORγ efficiently abolished the transcription of genes responsible for cholesterol synthesis and uptake while increasing cholesterol efflux. Notably, upon RORγ blockade the growth potential of control cells was preserved. Therefore, our results confirmed that RORγ serves as an activator of SREBP2-mediated cholesterol homeostasis not only in TNBC and prostate cancer but also for the first time in *t(4;11)* leukemia. By using specific antagonists in combination with chemotherapy or statins, leading to additive and synergistic effects, RORγ can be considered as a novel treatment strategy for *KMT2A*r leukemia.

## Results

### SREBP2 as master regulator of cholesterol pathway serves as possible target in *t(4;11)* leukemia

**Fig. 1:**
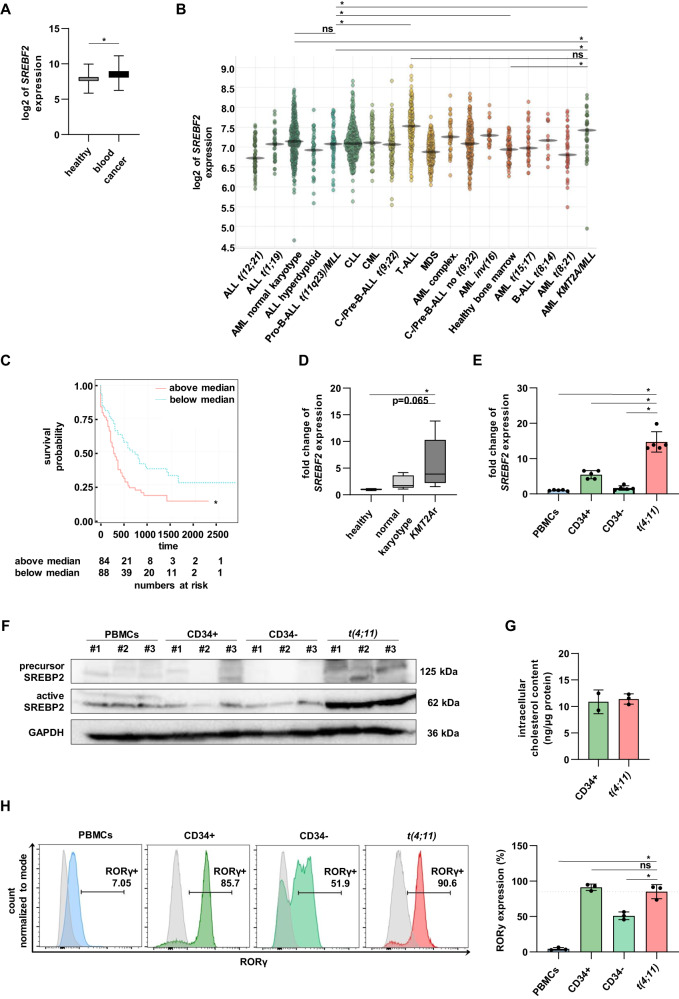
SREBP2 as master regulator of cholesterol pathway serves as potential target in *t(4;11)* leukemia. **A** log2 of *SREBF2* expression level of PBMCs from healthy patients (*n* = 915) and PBMCs from patients with blood cancer (*n* = 2082) using the gent2 database (http://gent2.appex.kr) with patient data from different datasets. Bars represent the mean ± standard deviation (SD). Student’s *t* test. *p < 0.05. **B** *SREBF2* expression in healthy and leukemic patient samples (GSE13159, data obtained from https://servers.binf.ku.dk/bloodspot/) [36]. The corresponding statistical analyses are shown in Supplementary Table 3. **C** Using data from AML patients from The Cancer Genome Atlas (https://tcga-data.nci.nih.gov/tcga), the overall survival of AML patients, divided into *SREBF2*^low^ (blue line) and *SREBF2*^high^ group (red line) according to the median value of *SREBF2* were compared with Kaplan–Meier analysis (data obtained from https://servers.binf.ku.dk/bloodspot/) [36]. Student’s *t* test. *p < 0.05. **D** Validation of *SREBF2* expression analyzed by RT-qPCR in PBMCs from patients with normal karyotype (*n* = 7) and patients with *KMT2A*r leukemia (*n* = 8) compared to PBMCs from healthy donors (*n* = 6). Bars represent the mean ± SD. One-way ANOVA. *p < 0.05. **E** *SREBF2* gene expression from RT-qPCR experiments. CD34+ HSPCs from huCB, CD34- cells differentiated over 3 weeks in myeloid culture from huCB-derived HSPCs and CRISPR/Cas9 *t(4;11)* cells were compared to PBMCs from healthy donors. Experiment was performed with *n* = 5 independent donors in technical duplicates with bars representing the mean ± SD. One-way ANOVA. *p < 0.05. **F** Representative Western blot analysis of full length and N-terminal SREBP2 in PBMCs from healthy donors, CD34+ HSPCs from huCB and CRISPR/Cas9 *t(4;11)* cells. Glyceraldehyde-3-phosphate dehydrogenase (GAPDH) was used as loading control. ^#^describes independent donors. **G** Total cellular cholesterol contents in CD34+ HSPCs huCB (*n* = 2) and CRISPR/Cas9 *t(4;11)* cells (n = 3) were analyzed with Amplex Red Assay and normalized to protein concentration. Data are shown as mean ± SD. **H** Representative flow cytometry histograms of intranuclear RORγ expression and pooled data from three independent donors (*n* = 3) performed in technical duplicates are shown. Bars representing the mean ± SD. One-way ANOVA was used. *p < 0.05. ns not significant.

In this study, we used publicly available patient databases to define the molecular role of SREBP2 in leukemia. To determine the overall *SREBF2* gene expression, gent2 database was used (which includes a wide range of independent patient datasets; see Supplementary Table 1). *SREBF2* gene expression was increased in patients with different blood cancer types compared to healthy individuals (Fig. 1A). An overview from *BloodSpot* showed the analysis of different acute leukemia subtypes with cytogenetic abnormalities using data from GSE13159 [36]. Here, the highest *SREBF2* expression was revealed in T-ALL followed by AML patients with *KMT2A*r (Fig. 1B, Supplementary Table 3). Additionally, cases with *KMT2A*r showed a significantly higher *SREBF2* expression than AML patients with normal karyotype and healthy bone marrow samples. In contrast, although expression in *KMT2A*r ALL patients was significantly higher than in healthy bone marrow samples, it was significantly lower than in patients with *KMT2A*r AML and T-ALL. To further assess the clinical relevance, AML cases of *The Cancer Genome Atlas* were divided into *SREBF2*^*l*ow^ and *SREBF2*^high^ groups. Overall survival rates of patients in the *SREBF2*^high^ group were significantly lower than those in the *SREBF*2^low^ group (Fig. 1C). To confirm these results in vitro, we analyzed gene expression in PBMCs from AML patients with normal karyotype (*n* = 7) and *KMT2A*r leukemia (*n* = 8) with RT-qPCR (Fig. 1D). Compared to PBMCs from healthy donors, both types of leukemia showed *SREBF2* significantly upregulated, interestingly with the highest trend in *KMT2A*r leukemia patients. Previously, we established a CRISPR/Cas9 *t(4;11)* leukemia model as a patient-like in vitro model [8]. We could confirm the significant higher *SREBF2* mRNA expression in our model compared to healthy PBMCs and CD34+ HSPCs derived from huCB (Fig. 1E). Interestingly, *SREBF2* expression strongly decreased when CD34+ HSPCs were expanded over 3 weeks in myeloid culture systems and lost their stem cell character (hereafter referred to as CD34−). The observed upregulation of *SREBF2* in *t(4;11)* leukemia was validated on protein level by Western blot analysis (Fig. 1F). Importantly, *t(4;11)* cells of our CRISPR/Cas9 model predominantly showed a high expression of the active, N-terminal form of SREBP2. Similar results were observed in *t(4;11)* cell lines (SEM, RS4;11, MV4-11) compared to SKM-1 at the mRNA (Supplementary Fig. 1A) and protein levels (Supplementary Fig. 1B). As SREBP2 serves as a master regulator of cholesterol homeostasis, we analyzed the intracellular cholesterol content in our CRISPR/Cas9 *t(4;11)* cells and freshly isolated CD34+ HSPCs (Fig. 1G). We did not detect any differences of the intracellular cholesterol level between the two groups and hypothesize that *t(4;11)* cells immediately consume excess of cholesterol to promote proliferation and to avoid an SREBP2-mediated negative feedback of sterol metabolism. As others reported, RORγ can act as possible upstream regulator of SREBP2 [33, 34]. Therefore, we measured RORγ expression in our model by intracellular staining and flow cytometry compared to different healthy cells. Similar to our findings with SREBP2, we detected a significantly higher RORγ expression in both CD34+ HSPCs and our CRISPR/Cas9 *t(4;11)* leukemia cells compared to PBMCs and differentiated HSPCs (CD34−) under myeloid culture systems (Fig. 1H). When we compared our results with public patient databases, we found a moderately higher expression of *RORC*, gene encoding RORγ, in *KMT2A*r leukemia patients (Supplementary Fig. 1C, Supplementary Table 4). Indeed, increased *RORC* expression did not correlate with poor survival of AML patients, indicating an independent role of *RORC* expression level and a functional role in the upregulation of SREBP2, which is responsible for an SREBP2-specific role in leukemia development (Supplementary Fig. 1D). These data indicate that SREBP2 as key regulator of cholesterol metabolism is highly overexpressed in *t(4;11)* leukemia and correlates with worse prognosis.

### Inhibition of mevalonate pathway induces antileukemic effects

**Fig. 2:**
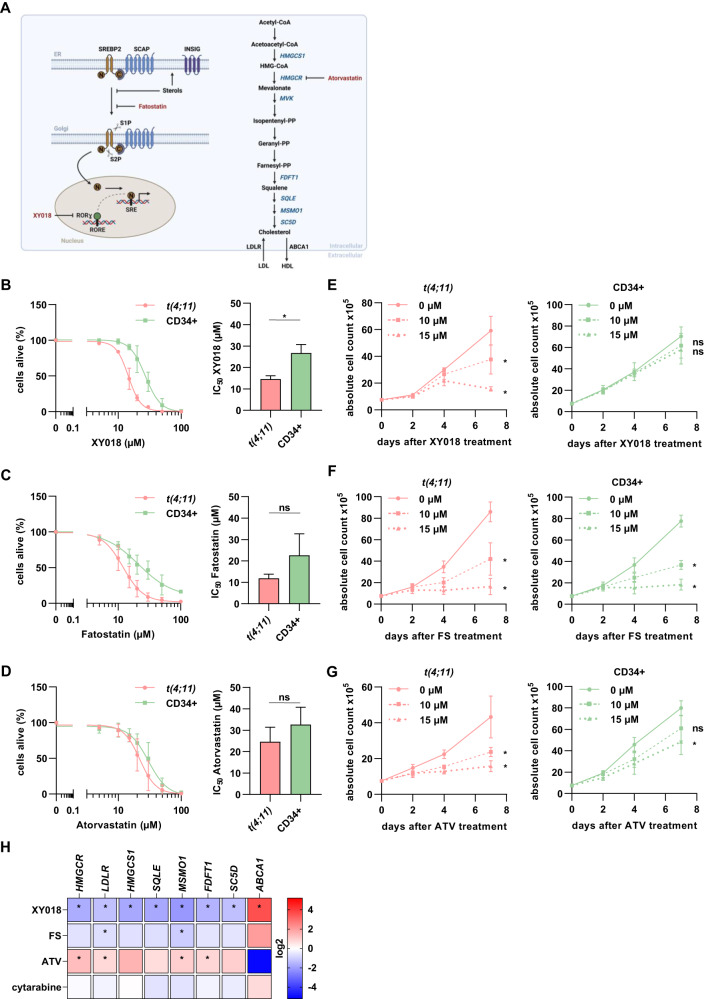
Inhibition of mevalonate pathway induces antileukemic effects. **A** Scheme representing the cholesterol pathway and its inhibition with different small molecules. CRISPR/Cas9 *t(4;11)* cells and CD34+ control cells (ctrl) were treated for 7 days with DMSO (0 µM) or increasing concentrations of **B** RORγ antagonist XY018, **C** SREBP2 inhibitor fatostatin (FS) or **D** HMGCR inhibitor atorvastatin (ATV). The percentage of living cells (annexin V−, propidium iodide [PI]−) was evaluated with flow cytometry and IC_50_ values were interpolated from a four-parameter logistic model constrained to 0 and 1 in GrapdhPad Prism. Cells were counted with trypan blue and absolute cell count was evaluated for different inhibitor concentrations (0, 10, 15 µM) for **E** XY018, **F** FS and **G** ATV for a total of 7 days. Experiments were performed with three independent donors (*n* = 3) in technical triplicates and dots represent the mean ± SD. One-way ANOVA. *p < 0.05. ns not significant. **H** Heat map display of fold changes (in log2) in genes of cholesterol homeostasis in CRISPR/Cas9 *t(4;11)* cells treated with 15 µM of each small molecule or 5 nM cytarabine for 7 days. The expression of indicated genes was measured by RT-qPCR for which DMSO-treated cells were set as 1. Experiments were performed with three independent donors (*n* = 3) in technical duplicates. One-way ANOVA. *p < 0.05.

Due to the observed strong expression of SREBP2 in *t(4;11)* leukemia cells, we further aimed to identify possible small molecules to inhibit downstream sterol homeostasis. For that, we selected three inhibitors with different modes of action on the cholesterol synthesis (Fig. 2A). We used XY018, a RORγ-selective antagonist [32], fatostatin (FS), which directly blocks SREBP2 by preventing its translocation to the Golgi apparatus [37] and atorvastatin (ATV), a widely used inhibitor of *HMGCR* to block cholesterol synthesis [23]. To define the cytotoxic profile and possible treatment side-effects, we used CD34+ cells with high levels of *SREBF2* mRNA and CD34- cells with low levels as control cells. First, we created dose-response profiles and IC_50_ values by increasing concentrations of the inhibitors, for which annexin V− and PI− cells were defined as alive. For the CRISPR/Cas9 *t(4;11)* cells treated with XY018, we revealed an IC_50_ value of 14.6 µM whereas CD34+ and CD34− control cells were significantly more robust (IC_50_ = 26.7 µM and 55.1 µM) (Fig. 2B, Supplementary Fig. 2A).

Similarly, FS and ATV induced a dose-dependent decrease in the percentage of living cells (Fig. 2C, D, Supplementary Fig. 2B, C). However, we did not find a therapeutic window as the control cells responded similarly to the respective treatment. By counting cells with trypan blue, the results showed a strong growth inhibition in CRISPR/Cas9 *t(4;11)* cells among 7 days of XY018 treatment (Fig. 2E). In contrast, we observed no growth inhibition effect on control cells using similar inhibitor concentrations. For validation of those effects, cell lines were treated with the RORγ antagonist XY018 as well and showed a significant sensitivity of *t(4;11)* cell lines (SEM, RS4;11 and MV4-11) compared to the non-translocated cell line SKM-1 (Supplementary Fig. 2D, E). In contrast, FS and ATV induced almost similar leukemic and healthy cell death (Fig. 2F, G). Due to the controversial results of the different small molecules, we were highly interested in their effect on key cholesterol biosynthesis genes in *t(4;11)* leukemia. Therefore, mRNA levels of cholesterol-related target genes were measured by RT-qPCR 7 days after treatment with the respective inhibitors. As expected, treatment of FS clearly reduced the mRNA expression of cholesterol-related target genes and increased cholesterol efflux by *ABCA1* transcription (Fig. 2H). Consistent with previous studies on solid tumors [30, 35], addition of ATV strongly increased cholesterol biosynthesis genes while decreasing *ABCA1* due to the known statin-induced feedback. Interestingly, the effects of the RORγ inhibitor on cholesterol-related target genes were comparable with the direct SREBP2 inhibitor FS but much more pronounced. Moreover, XY018 treatment had much less influence on the mRNA level of these genes in control cells (Supplementary Fig. 2F) or in the *t(4;11)*-negative cell line SKM-1 (Supplementary Fig. 2G), with the exception of *ABCA1*, whose alteration, however, had no negative effect on cell proliferation or viability. These data implicate that inhibition of cholesterol metabolism by XY018 leads to specific antileukemic effects in *t(4;11)* leukemia and that cholesterol metabolism correlates with cell growth hereby promoting *KMT2A*r leukemogenesis.

### Inhibition of RORγ reduces cell viability by induced apoptosis and changes cell cycle of *t(4;11)* leukemia cells

**Fig. 3:**
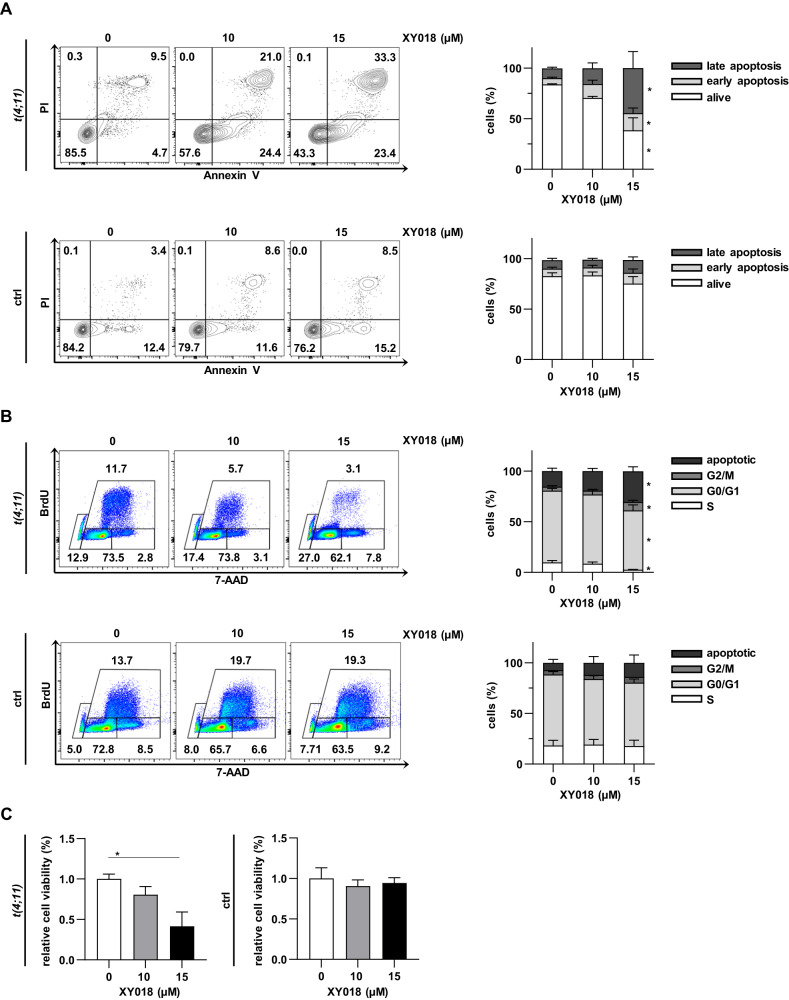
Inhibition of RORγ reduces cell viability by induced apoptosis and changes cell cycle of *t(4;11)* leukemia cells. **A** Representative dot plots and pooled data of CRISPR/Cas9 *t(4;11)* and CD34+ cells (ctrl) showing living (annexin V−, PI−), early apoptotic (annexin V+, PI−) and late apoptotic cells (annexin V+, PI+). Cells were treated with indicated concentrations of XY018 for 7 days. Histograms show the mean of three independent donors (*n* = 3) ± SD. One-way ANOVA. *p < 0.05. **B** CRISPR/Cas9 *t(4;11)* and CD34+ cells (ctrl) were treated with different concentrations of XY018 for 7 days and analyzed for cell cycle distribution using BrdU staining and flow cytometry. Histograms show the mean of three independent donors (*n* = 3) ± SD. One-way ANOVA. *p < 0.05. **C** Cells treated with XY018 or DMSO were analyzed for cell viability on day seven of treatment using alamarBlue assay. Bars represent the mean of three independent donors (*n* = 3) ± SD. One-way ANOVA. *p < 0.05.

Since the RORγ antagonist XY018 had a negative impact on proliferation in *KMT2A*r cells, we further investigated the consequences of cholesterol pathway inhibition on apoptosis and cell cycle. Upon XY018 treatment, CRISPR/Cas9 *t(4;11)* cells showed a significant increase of early and late apoptotic cells whereas healthy HSPCs were not affected (Fig. 3A). These findings were confirmed by staining with 5′-bromo-2′-deoxyuridine (BrdU) and 7-amino-actinomycin D (7-AAD) showing a diminished occurrence of cells in S and G0/G1 phase. Simultaneously, we observed an XY018 concentration-dependent increase of the frequency of arrested cells in G2/M phase, whereas again the control cells were not affected (Fig. 3B). Since inhibition of cholesterol pathway is necessarily associated with metabolic activity, CRISPR/Cas9 *t(4;11)* cells exhibited a reduced cellular viability after 7 days of XY018 treatment measured by the alamarBlue cell viability assay (Fig. 3C). Here, similar results with regard to cell cycle distribution and apoptosis were observed in *t(4;11)* cell lines, whereas SKM-1 cells were almost not affected upon RORγ inhibition (Supplementary Fig. 3A–C). These data suggest that the inhibition of RORγ results in induction of cell cycle arrest and finally apoptosis of *KMT2A*r fusion-driven leukemia without any negative impact on control cells.

### RORγ controls the cholesterol-dependent gene program and is an upstream regulator of SREBP2 in *t(4;11)* leukemia

**Fig. 4:**
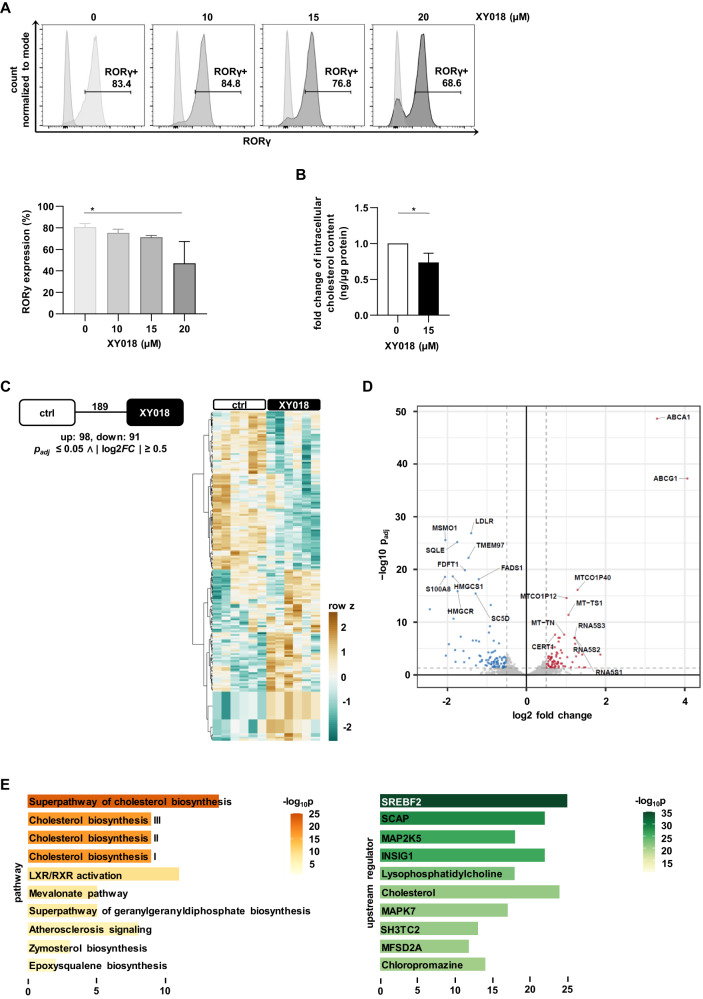
RORγ controls the cholesterol-dependent gene program and is an upstream regulator of SREBP2 in *t(4;11)* leukemia. **A** Representative histograms and pooled data show the percentage of intranuclear RORγ positive cells analyzed with flow cytometry. CRISPR/Cas9 *t(4;11)* cells were treated with DMSO or indicated concentrations of XY018 (10 µM, 15 µM and 20 µM) for 7 days. Bars represent the mean of three independent donors (*n* = 3) ± SD. One-way ANOVA. *p < 0.05. **B** Total cellular cholesterol contents of CRISPR/Cas9 *t(4;11)* cells treated with DMSO or 15 µM XY018 for 7 days were measured with Amplex Red after organic extraction and normalized to protein concentration. DMSO-treated cells were set as 1. Bars represent the mean of three independent donors (*n* = 3) ± SD. Student’s *t* test. *p < 0.05. **C** CRISPR/Cas9 *t(4;11)* cells were treated with DMSO (control) or 15 µM XY018 for 7 days. Three independent donors (*n* = 3) in biological duplicates were used for RNA-Seq. Analysis revealed 189 differentially expressed genes (DEGs). Their expression profile across all samples is shown as z-score. **D** Volcano plot highlighting 91 downregulated (blue) and 98 upregulated (red) DEGs. Dotted lines indicate significance thresholds (*p*_FDR_ ≤ 0.05, |log_2_(fold-change)| ≥ 0.5). **E** Top ten overrepresented pathways and predicted upstream regulators among all DEGs according to Ingenuity Pathway Analysis (IPA) with gene count and significance values in bar graphs.

Recently, it has been shown that the newly developed RORγ-antagonist, XY018, was able to inhibit the growth of androgen receptor-positive prostate cancer via downregulation of RORγ in tumor cells [32]. To confirm the inhibitory effect of XY018 in *t(4;11)* leukemia, we measured the expression of RORγ in the CRISPR/Cas9 *t(4;11)* cells upon increasing concentrations of XY018 with intracellular flow cytometry and found an analogous dose-dependent decrease of the target protein (Fig. 4A). Next, we determined the intracellular cholesterol level in *t(4;11)* cells treated with XY018 or DMSO and found, as expected, a significant reduction in intracellular cholesterol (Fig. 4B). Finally, we performed RNA sequencing (RNA-Seq) of XY018-treated CRISPR/Cas9 *t(4;11)* cells to identify the core transcriptional program that is controlled by RORγ. This revealed 189 differentially expressed genes (DEGs) with 98 genes upregulated and 91 genes downregulated (Fig. 4C, Supplementary Table 3, Supplementary Table 4). The top ranked downregulated genes are mainly associated with cholesterol uptake and synthesis (*LDLR*, *TMEM97*, *MSMO1*, *SQLE*, *FDFT1*, *HMGCS1*, *HMGCR*, *SC5D*). In contrast, the most highly expressed genes after RORγ inhibition are linked to cholesterol efflux (*ABCA1*, *ABCG1*) (Fig. 4D). Furthermore, Ingenuity Pathway Analysis (IPA) revealed that RORγ inhibition resulted in the downregulation of several sub-pathways of cholesterol metabolism in *t(4;11)* cells. Upstream transcription factor analysis predicted, at first place, the function of *SREBF2* most significantly decreased in response to treatment with the antagonist (Fig. 4E). RNA-Seq data have been deposited in NCBI’s Gene Expression Omnibus (GEO) and are accessible through GEO Series accession number GSE242401. These results demonstrate that XY018 leads to a specific alteration of cholesterol biosynthesis genes and confirm that RORγ plays a prominent role in terms of the function of SREBP2 in *t(4;11)* leukemia.

### RORγ inhibition sensitizes CRISPR/Cas9 *t(4;11)* cells to chemotherapy and acts synergistically in combination with ATV

**Fig. 5:**
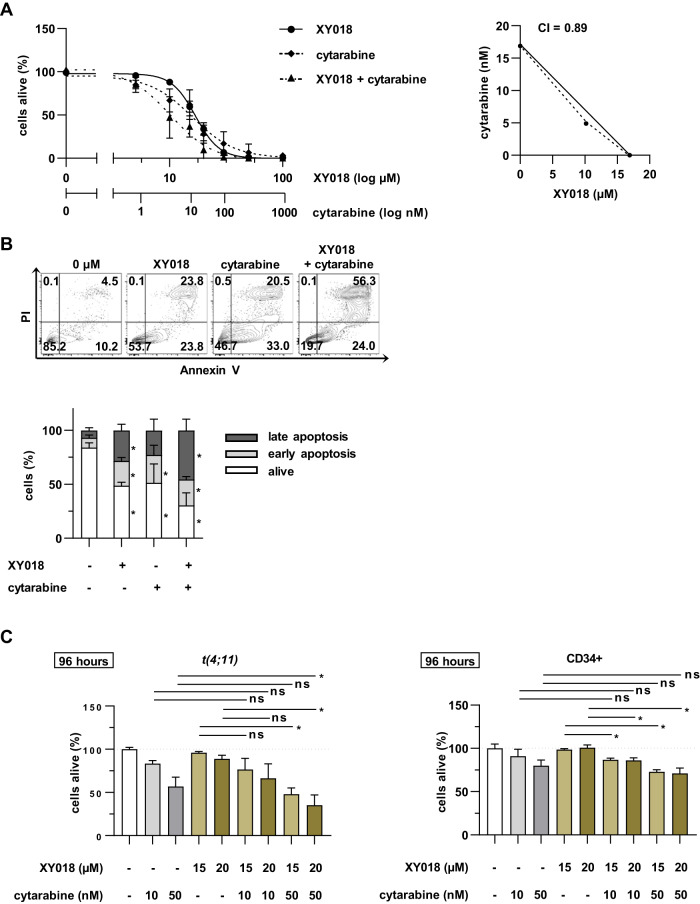
RORγ inhibition sensitizes CRISPR/Cas9 *t(4;11)* cells to chemotherapy. **A** CRISPR/Cas9 *t(4;11)* cells were simultaneously treated with increasing concentrations of XY018 alone or in combination with cytarabine for 7 days. The percentage of living cells (annexin V−, PI−) was evaluated with flow cytometry. IC_50_ values were mapped as isobologram and the Chou-Talalay method was used to measure the CI for identification of synergistic, additive or antagonistic effects. **B** For measurement of apoptosis, CRISPR/Cas9 *t(4;11)* cells were treated with 15 µM XY018 alone or in combination with 10 nM cytarabine. DMSO-treated or single-treated cells were used as control, respectively. Representative dot plots (top) and pooled data (bottom) show different apoptotic stages of three independent donors (*n* = 3) measured in technical duplicates. Bars represent the mean ± SD. One-way ANOVA. *p < 0.05. **C** CRISPR/Cas9 *t(4;11)* and CD34+ control cells were treated with 15 µM or 20 µM XY018 alone or in combination with 10 nM or 50 nM cytarabine for a total of 96 h. The percentage of living cells (annexin V−, PI−) was evaluated with flow cytometry, and vehicle-treated cells were set to 100 as an internal control. Bars represent the mean of three independent donors (*n* = 3) ± SD. One-way ANOVA. *p < 0.05. ns not significant.

**Fig. 6:**
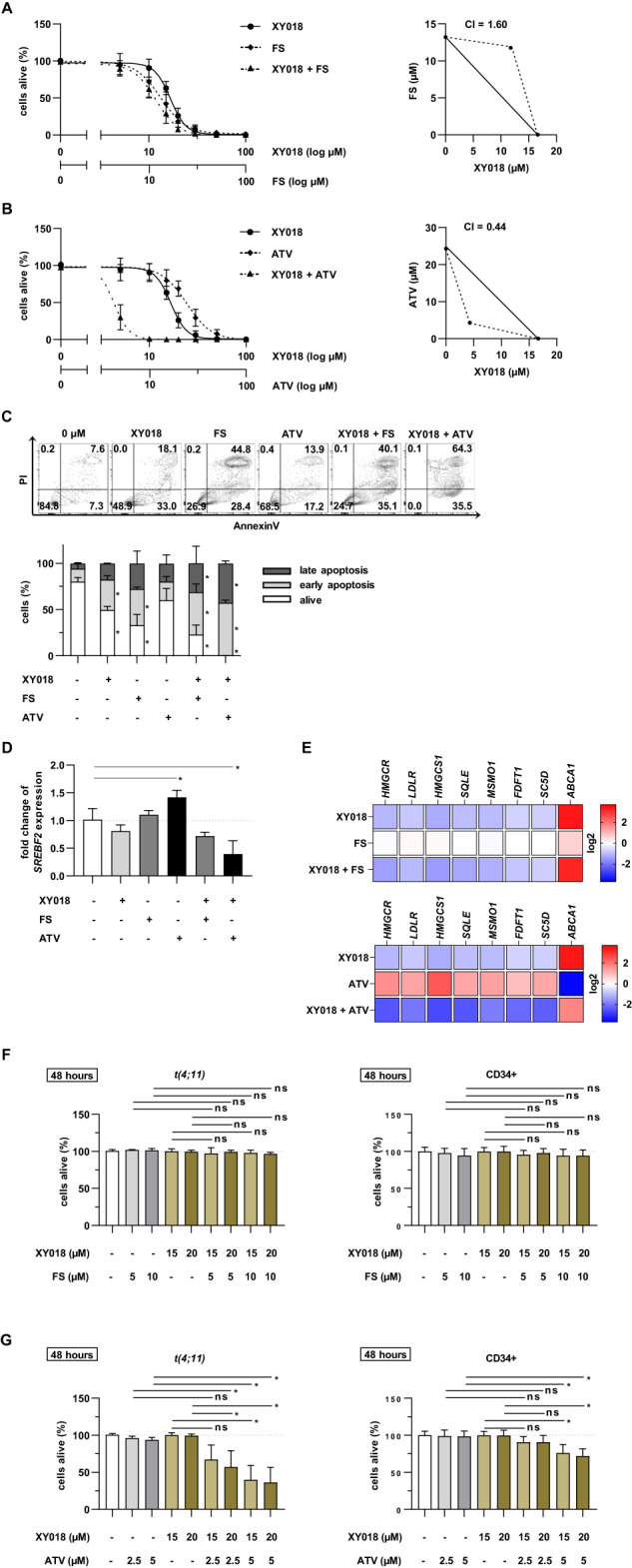
RORγ inhibition in CRISPR/Cas9 *t(4;11)* cells acts synergistically in combination with ATV. CRISPR/Cas9 *t(4;11)* cells were simultaneously treated with increasing concentrations of XY018 alone or in combination with FS (**A**) or ATV (**B**) for 7 days. The percentage of living cells (annexin V−, PI−) was evaluated with flow cytometry. IC_50_ values were mapped as isobologram and the Chou-Talalay method was used to measure the CI for identification of synergistic, additive or antagonistic effects. **C** Apoptosis was measured in CRISPR/Cas9 *t(4;11)* cells treated with 15 µM XY018 alone or in combination with 15 µM FS or ATV. DMSO-treated or single-treated cells were used as control, respectively. Representative dot plots (top) and pooled data (bottom) show different apoptotic stages of three independent donors (*n* = 3) measured in technical duplicates. Bars represent the mean ± SD. One-way ANOVA. *p < 0.05. **D** RT-qPCR analysis of *SREBF2* gene expression in the CRISPR/Cas9 *t(4;11)* cells treated with 15 µM XY018, 15 µM FS or 15 µM ATV alone or in combination for 72 h. Bars represent the mean of three independent donors (*n* = 3) ± SD. One-way ANOVA. *p < 0.05. **E** Heat map display of fold changes (in log2) in genes of cholesterol homeostasis in CRISPR/Cas9 *t(4;11)* cells treated as described in (**D**). The expression of indicated genes was measured by RT-qPCR for which DMSO-treated cells were set as 1. Experiments were performed with three independent donors (*n* = 3) in technical duplicates. One-way ANOVA. *p < 0.05. CRISPR/Cas9 *t(4;11)* and CD34+ control cells were treated with indicated concentrations of XY018 alone or in combination with FS (**F**) and ATV (**G**) for a total of 48 h. The percentage of living cells (annexin V−, PI−) was evaluated with flow cytometry, and vehicle-treated cells were set to 100 as an internal control. Bars represent the mean of three independent donors (*n* = 3) ± SD. One-way ANOVA. *p < 0.05. ns=not significant.

Cytarabine is extensively used as chemotherapeutic drug for the treatment of AML. Therefore, we wanted to assess the potential of RORγ inhibition to improve the efficacy of chemotherapy in a co-treatment approach. Interestingly, combination of XY018 and cytarabine induced additive effects regarding cell survival (Fig. 5A). Furthermore, simultaneously treated CRISPR/Cas9 *t(4;11)* cells showed significantly increased apoptosis compared to the single treatment (Fig. 5B). To determine a therapeutic window, CRISPR/Cas9 *t(4;11)*, cell lines and control cells were treated with the same combinations, showing a stronger effect in the leukemic cells (Fig. 5C, Supplementary Fig. 4A, B). Next, we examined the therapeutic potential of the RORγ antagonist XY018 in combination with FS and ATV. CRISPR/Cas9 *t(4;11)* cells were treated with increasing concentrations of FS and ATV alone or in combination with XY018. To define drug combination according to Chou-Talalay [38], we created dose-response curves of the inhibitors alone or in combination and plotted IC_50_ values in isobolograms. In the case of combining XY018 with FS, we observed a clear antagonistic effect on the percentage of living cells (Fig. 6A), whereas the combination of XY018 and ATV increased cell death synergistically (Fig. 6B). Assessment of apoptosis did not show an improved effect upon combining XY018 and FS but the combination of XY018 and ATV significantly caused pronounced apoptosis as reflected by annexin V assay (Fig. 6C). We further investigated the effects of both combinations on *SREBF2* gene expression. The results showed a significantly increased *SREBF2* gene expression in ATV-treated cells and a significant downregulation by combining XY018 and ATV (Fig. 6D). We confirmed the results by RT-qPCR of cholesterol-related target genes, where the combination of XY018 and FS did not show a difference in *SREBF2* expression compared to the single treatment. Indeed, treatment of cells with the RORγ antagonist reversed the statin-induced upregulation of cholesterol biosynthesis resulting in a superior inhibition of those key genes (Fig. 6E). Additionally, the direct comparison of healthy and leukemia cells confirmed the effect of XY018 in combination with FS, which did not confer any benefit (Fig. 6F, Supplementary Fig. 4C). When XY018 and ATV were combined, synergistic effects of the two drugs were seen in all cells. But the effects were most pronounced in the CRISPR/Cas *t(4;11)* cells and *t(4;11)* cell lines, suggesting a therapeutic window for the treatment of *t(4;11)* leukemia (Fig. 6E, Supplementary Fig. 4D). Taken together, these data provide evidence that XY018 can be beneficially combined with both chemotherapy and statins.

## Discussion

For a variety of cancer cells it is known that they change their cholesterol homeostasis to increase proliferation and survival [12, 39]. Indeed, SREBPs have been implicated as important metabolic transcription factors in several cancers. Among its specific role in regulating mevalonate pathway, there is growing evidence that activation of SREBP2 is necessary to ensure cancer cell proliferation. For instance, downregulation of SREBPs inhibited colon cancer cell proliferation and in vivo tumorigenesis as a result of decreased fatty acid and cholesterol synthesis [40]. However, until now, the role of SREBP2 in leukemogenesis and its relevance as therapeutic target in *KMT2A*r leukemia, a subtype of leukemia with specifically poor prognosis, has not been investigated. In this study, we showed that especially *t(4;11)* leukemia cells exhibited an activated SREBP2-dependent cholesterol homeostasis justifying the high proliferation index. Therefore, we uncovered the critical function of SREBP2 and the nuclear receptor RORγ in both the maintenance of leukemic cell growth in vitro and as promising therapeutic targets in *t(4;11)* leukemia.

Early observations from Vitols et al. show that AML cells exhibit high rates of cholesterol import and synthesis. In this context, *HMGCR* as a fundamental metabolic gene has been characterized very well [20, 41–43]. These studies are also in line with the common understanding that increased cholesterol levels may serve to protect leukemic cells. Here, we provide data that *t(4;11)* leukemia cells upregulated their metabolism at a much earlier time point, which is at the transcriptional level and not only during cholesterol synthesis. Moreover, besides our *t(4;11)* leukemia model, we confirmed the increased SREBP2 activity in *t(4;11)* cell lines, primary cells and publicly available patient data. Of note, compared to patient data where *KMT2A*r AML cells had higher SREBP2 expression than *KMT2A*r ALL cells, the opposite was true for the commercially available *KMT2A*r cell lines. Although the expression level of *KMT2A*r cell lines generally resulted in a correspondingly higher sensitivity to XY018 treatment, the differences between patient material and commercially available cell lines are already known. Therefore, in this study, we mainly successfully used our patient-derived CRISPR/Cas9 model to decipher the importance of SREBP2 in leukemogenesis and as a therapeutic target.

Strikingly, the expression level of *SREBF2* correlated with the overall survival in leukemic patients demonstrating again the major role of *SREBF2* as signaling pathway in this disease [36]. Controversially, the level of expression of *RORC* did not correlate with overall survival, suggesting an independent role of the expression level. Contrary to the results of others, we did not find increased intracellular cholesterol levels compared to healthy cells. We claim that *t(4;11)* cells are able to directly consume excess of cholesterol for cell growth and thus prevent the feedback response regarding reduced SREBP2 activity.

As cholesterol metabolism is regulated in multi-levels by cross-linked pathways, we used different small molecules to inhibit the metabolic activity of *t(4;11)* cells. As expected, we found a significant decrease of cholesterol synthesis and uptake genes upon treating cells with FS, a direct SREBP2 inhibitor that changes cholesterol gene expression by disrupting the nuclear translocation of SREBP2. Nevertheless, as also reported from others, FS had toxic potential and also inhibited cellular growth of our control cells [44]. Upon treatment with ATV, we observed an upregulation of *SREBF2* at the transcriptional level and consequently of target genes such as *HMGCR*, *HMGCS1* or *LDLR*. Surprisingly and contrary to our results showing an upregulated cholesterol pathway at the mRNA level, both *t(4;11)* and control cells exhibit a reduction in cellular growth. We explain our results by the observation of another group showing that statins are also able to induce intrinsic apoptotic pathways in most cancer cells by disrupting the isoprenylation of Rho family and other key regulatory proteins [45, 46]. Therefore, it seems obvious that the anti-proliferative effects of statins on *t(4;11)* cells are also not only due to inhibition of cholesterol metabolism but to activation of pro-apoptotic pathway proteins [31, 47]. Although it is generally safe to use statins at doses for treatment of hypercholesterolemia, their efficacy as potential anticancer treatment in clinical studies is limited and less encouraging [27, 28, 48]. An explanation is, comparable to our findings, that statin-induced inhibition of HMGCR leads to feedback activation of SREBP2 and subsequently to increased LDLR-mediated cholesterol uptake and synthesis. This makes cancer therapy less effective [29, 49]. Therefore, therapeutic strategies avoiding this negative feedback are required.

However, the key regulator of sterol metabolism, SREBP2, is a highly conserved transcription factor and difficult to target. But findings of others regarding solid tumors have brought RORγ into focus as new known driver of cholesterol metabolism, which is located upstream and directly regulates SREBP2. Recently, a new inhibitor of RORγ, named XY018, enables the efficient blockade of the cholesterol metabolism without activation of the negative feedback and promotes anti-tumor effects [34]. By using XY018 in our study, we observed that inhibition of RORγ induced leukemic cell death, apoptosis and significant cell cycle arrest of *t(4;11)* cells. Interestingly, effective targeting of RORγ by XY018 resulted in a much stronger inhibition of cholesterol pathway-related genes than treatment with FS. We therefore conclude that direct inhibition of RORγ, and thus indirect inhibition of the transcriptional action of SREBP2, is a more promising therapeutic approach than direct inhibition by FS, which also leads to unknown toxicity. Similarly, RORγ was shown to act as potent transcriptional activator in some cancer cells. Others were able to prove a potent RORγ interaction with SREBP2 at the chromatin level leading to a hyper-stimulated cholesterol homeostasis in TNBC [34, 50]. In prostate cancer, the nuclear receptor suppresses the expression of cholesterol efflux genes under pro-tumor conditions while treatment with the RORγ antagonist abolished statin-induced, SREBP2-mediated feedback [35]. In our study, we compared the gene expression profiles by RNA-Seq before and after RORγ inhibition and identified RORγ as an activator of SREBP2-mediated gene program in *t(4;11)* cells as well. Indeed, we also observed the strongest upregulation after RORγ inhibition on cholesterol efflux genes (*ABCA1*, *ABCG1*) confirming its role as repressor to silence liver X receptor (LXR) downstream genes. Although the total number of deregulated genes was low and mainly focused on cholesterol metabolism, suggesting specificity of the drug, we also detected increased expression of genes encoding ribosomal 16S. We interpreted this as a possible compensatory mechanism of cells for cholesterol deficiency [51]. In addition, some mitochondrial genes known to be characteristic of early stages of apoptosis were upregulated, consistent with the observed apoptotic phenotype of our cells after inhibition [52]. A more detailed analysis of this phenomenon isneeded to investigate possible side-effects on mitochondria, which are known to require cholesterol for their maintenance.

Since also healthy cells require cholesterol for their proliferation and especially immature cells harbor a high SREBP2 and RORγ expression, we investigated the sensitivity of CD34+ HSPCs upon RORγ inhibition. While *t(4;11)* cells underwent apoptosis and cell cycle arrest, CD34+ HSPCs were nearly resistant under equal conditions. This was further confirmed by using another control with CD34+ cells cultured for 3 weeks under myeloid conditions (CD34−), which had lower levels of SREBP2. Consistent with the level of SREPB2, these cells were even less sensitive to RORγ inhibition, demonstrating SREBP2 dependence. Moreover, Cai et al. could demonstrate that XY018-treated mice did not exhibit relevant side-effects, in particular, liver function and blood components including immune cells were not altered after treatment in vivo [34]. These results offer a potential therapeutic window for the translation of our data into a clinical setting. Notably, we also observed a higher sensitivity of *t(4;11)* cell lines compared to a non-*KMT2A*r cell line, highlighting the specificity of the studied pathway and the importance to define special subgroups of patients who might benefit from the therapy.

Since chemotherapy is the standard treatment for leukemia patients, the interactions of such agents on the cholesterol metabolism has been also investigated in vitro. For instance, it has been shown that elevated cholesterol levels are associated with resistance to common induction chemotherapies such as daunorubicin and cytarabine [16]. Interestingly, the antileukemic effect of decitabine has been reported to be mechanistically linked to the inhibition of cholesterol metabolism [14] and, moreover, cholesterol level reducing drugs induced cytotoxic effects in AML stem cells [24, 53]. Furthermore, AML cell lines respond to chemotherapeutics by increasing their intracellular cholesterol levels, and in turn, primary AML cell samples could be sensitized to cytarabine by mevastatin [16, 24]. However, in our setting the *t(4;11)* cells did not respond to chemotherapy exposure with an upregulated cholesterol target gene expression. Nevertheless, in a combinatorial treatment experiment with cytarabine and XY018, we found a beneficial outcome by additive effects on reduced *t(4;11)* cell viability. Even more interesting is our observation that inhibition of RORγ in combination with ATV, an agent that is widely used and overall well-tolerated in the context of cardiovascular diseases, significantly reduced *SREBF2* gene expression. At the same time, the statin-induced feedback response was completely abolished, resulting in a potent anti-leukemia synergism. Our results can be improved by the results of studies with solid cancer cells which have been provided nearly identical findings regarding the synergistic effect by combining XY018 and statins [34, 35]. Although no in vivo experiments were performed in our study that could lead to limitations of the conclusions drawn here, we are convinced that our in vitro experiments with the patient-like CRISPR/Cas9 *t(4;11)* model contribute to give a more comprehensive view of the association between cancer and cholesterol pathway as a general targeting mechanism for rapidly growing tumor cells.

Taken together, our in vitro experiments provide the rationale that metabolic dysregulations play a major role in the pathogenicity of *KMT2A*r patients, especially with *t(4;11)* leukemia. In this context, RORγ serves as promising target to promote antileukemic effects, which can be combined with chemotherapies and statins. Thus, limitations regarding the negative statin-induced feedback can be circumvented and therapeutic efficacy can be enhanced to eventually overcome the poor prognosis of *KMT2A*r leukemia patients.

## Methods

### Human CRISPR/Cas9 *t(4;11)* model generation

CD34+ hematopoietic stem and progenitor cells (HSPCs) were isolated from fresh human umbilical cord blood (huCB) obtained from the Department of gynecology from the University Hospital Tuebingen (IRB approvals 751/2015BO2 and 461/2022BO2) using Ficoll Paque (PAN-Biotech GmbH, Aidenbach, Germany) followed by the isolation of HSPCs with the human CD34+ Microbead Kit (Miltenyi, Bergisch Gladbach, Germany). *t(4;11)* was induced using an RNP complex of specific self-made sgRNAs and Cas9 protein (PNA Bio Inc., Thousand Oaks, CA, USA) as previously described [8, 54]. A pure culture of *t(4;11)* cells was defined by more than 90% of translocated cells determined by Fluorescence-In-Situ-Hybridization (FISH) as previously described [54]. CD34+ and *t(4;11)* cells were maintained in StemMACS™ HSC Expansion Media (Miltenyi) supplemented with 10% FCS (Gibco by Thermo Fisher Scientific, Waltham, MA, USA), 1% Penicillin/Streptomycin (Lonza, Basel, Switzerland), human cytokines (IL-3, IL-6, SCF, FLT3L, SCF, G-CSF, 50 ng/ml each, PeproTech, Rocky Hill, NJ, USA), SR-1 and UM-729 (0.75 µM each, STEMCELL Technologies, Vancouver, Canada).

### Chemicals

RORγ inhibitor XY018 (purity >98%) was obtained from Tocris Bioscience (Bristol, UK). SREBP2 inhibitor fatostatin (purity >99%) and HMGCR inhibitor atorvastatin (purity >98%) were obtained from MedChemExpress (Monmouth Junction, NJ, USA) and Selleck Chemicals (Houston, TX, USA). Cytarabine was obtained from Stadapharm (Bad Vilbel, Germany).

### Single inhibitor treatment, cell count and viability

*t(4;11)* and CD34+ control cells were seeded in 48- or 96-well plates at a density of 7.5 × 10^5^/ml in appropriate media and cultured for a maximum of 7 days. Compounds were prepared in stock solutions with DMSO and added to the cells in serial dilution on day 0. Cells were reseeded in the origin density and retreated with inhibitors after 48 h and 72 h.

SKM-1, SEM and RS4;11 cells were seeded at a density of 5 × 10^5^/ml and MV4-11 with 2 × 10^5^/ml in their appropriate media and treated with inhibitors. Cell lines were reseeded and retreated after 48 h and analyzed after 72 h.

For proliferation curves over time, total viable cell numbers were counted with 0.04% trypan blue (Merck, Darmstadt, Germany). Cell viability was assessed by adding alamarBlue cell viability reagent (Thermo Fisher Scientific) as previously described [9].

### Combinatorial treatment studies

For combinatorial treatment studies, cells were treated with different concentrations of the respective compounds alone or in combination for a total of 7 days. The data are presented as percentage of viable cells (defined as propidium iodide and annexin negative) with DMSO-treated cells set as 100%. The estimated in vitro IC_50_ values were calculated using GraphPad Prism 9 software (v9.3.1, GraphPad Software Inc., San Diego, CA, USA). Combination index (CI) was analyzed by the Chou–Talalay method [38] and used to define synergy (CI < 1), additivity (CI = 1) and antagonism (CI > 1).

### Measurement of intracellular cholesterol content

1 × 10^6^ cells were washed with cold PBS and extracted in methanol/chloroform/ddH_2_O (2:2:1) followed by incubation on a shaker for 25 min at room temperature. After incubation, the homogenized solution was centrifuged at 800 × *g* for 15 min. The organic phase was collected, dried under nitrogen supply and lipids were resuspended in 400 µl 1× reaction buffer contained in the Amplex® Red Cholesterol Assay kit (Thermo Fisher Scientific). Intracellular cholesterol content was measured in triplicates according to the manufacturer’s protocol and normalized to protein concentrations.

### Supplementary information

Erkner et al. Supplemental Material

## Data Availability

The datasets generated during and/or analyzed during the current study are available from the corresponding author on reasonable request.
