# Supplementary material for: The RORɣ/SREBP2 pathway is a master regulator of cholesterol metabolism and serves as potential therapeutic target in t(4;11) leukemia
Source: Oncogene. 2023 Nov 29;43(4):281–93. doi: 10.1038/s41388-023-02903-3 (PMC10798886; doi:10.1038/s41388-023-02903-3)
Supplement: Supplementary file 1 — Erkner et al. Supplemental Material [file 41388_2023_2903_MOESM1_ESM.pdf]

Supplementary Materials

The RORɣ/SREBP2 pathway is a master regulator of cholesterol metabolism and serves as potential therapeutic target in *t(4;11)* leukemia

Estelle Erkner, Thomas Hentrich, Rebekka Schairer, Rahel Fitzel, Kathy-Ann Secker-Grob, Johan Jeong, Hildegard Keppeler, Fulya Korkmaz, Julia M. Schulze-Hentrich, Claudia Lengerke, Dominik Schneidawind, Corina Schneidawind

Supplementary Methods

Cell culture/ cell lines

RS4;11 and MV4-11 cells were cultured in RPMI1640 (Gibco by Thermo Fisher Scientific, Waltham, MA, USA) supplemented with 10% FCS and 1% penicillin/streptomycin. SEM cells were grown in IMDM (Gibco) supplemented with 10% FCS and 1% penicillin/streptomycin. SKM-1 cells were cultured in RPMI1640 supplemented with 15% FCS and 1% penicillin/streptomycin. The listed cell have recently been authenticated and tested negative for mycoplasma contamination.

Patient samples

Peripheral blood mononuclear cells (PBMCs) from patients with *KMT2A*r leukemia were isolated after written informed consent had been obtained and were performed by the University Children's Hospital Tuebingen. The study was approved by our Institutional Review Board to be in accordance with ethical standards and with the Helsinki Declaration of 1975, as revised in 2013 (IRB approval 137/2017BO2).

RNA isolation, cDNA synthesis, RT-qPCR

Total RNA was isolated from cells using NucleoSpin RNA Kit (Macherey-Nagel, Dueren, Germany) and the cDNA was prepared with RevertAid H Minus Reverse Transcriptase, RiboLock RNase Inhibitor, dNTP Mix, and Random Hexamers (all from Thermo Fisher Scientific) according to the manufacturer's protocols. RT-qPCR was performed as previously described (1) using a LightCycler 480 Instrument II (Roche, Basel, Switzerland). The fluorescence signals were collected, and fold difference was calculated employing the ddCT method. 18S rRNA was used as the internal reference to normalize the relative level of each transcript. The experiments were performed at

least three times with data represented as mean values ± SD. Primer sequences are listed in Supp. Table 2.

Western blot

Whole cell lysates were prepared using RIPA buffer containing protease inhibitors and protein concentration was evaluated with DC Assay (BioRad, Hercules, CA, USA) according to the manufacturer's protocol. An equal protein amount was denatured in Laemmli buffer, separated on a 10% SDS gel and transferred to an Immobilon-FL PVDF membrane (Merck, Darmstadt, Germany). Membranes were blocked with non-fat dry milk, washed with TBS buffer containing 0.2 % tween 20 and incubated overnight with primary antibodies against SREBP2 (Abcam, Cambridge, UK, 1:500) and GAPDH (Cell Signaling Technology, Danvers, MA, USA, 1:1000). Membranes were washed and incubated with secondary antibody (Cell Signaling Technology, anti-rabbit IgG, HRP-linked). The signal was developed using the the Pierce™ ECL Western Blotting Substrate (Thermo Fisher Scientific) and visualized in the Fusion FX (Vilber Lourmat, Eberhardzell, Germany).

Intracellular protein expression via flow cytometry

For antibody staining, cells were washed with flow buffer (PBS with 2% FCS) and incubated with human Fc Receptor Binding Inhibitor (Miltenyi, Bergisch Gladbach, Germany, 1:100) for 5 min at room temperature. All following staining steps were performed at 4 °C and in the dark. Cells were stained with viability dye eFluor506 (Thermo Fisher Scientific, 1:1000) for 20 min. Thereafter, cells were fixed and permeabilized with eBioscience™ Foxp3/Transcription Factor Staining Buffer Set (Thermo Fisher Scientific) for 60 min before being washed twice with 1x permeabilization buffer. Intracellular RORγ staining was performed for 1h using anti-

RORγ-APC (Thermo Fisher Scientific, 1:200). Samples were washed twice with 1x permeabilization buffer and stored at 4 °C until acquisition. At least, 30,000 viable cells per sample were acquired for analysis using an LSR Fortessa (BD Biosciences, San Jose, CA, USA). Data were analyzed using FlowJo V10 software (BD Biosciences).

Apoptosis and cell cycle

For apoptosis and cell cycle assays, cells were analyzed using FITC Annexin V Apoptosis Detection Kit I (Miltenyi) and FITC BrdU Flow Kit (BD Biosciences) according to the manufacturer's protocols.

RNA library preparation, sequencing and data analysis

Total RNA was isolated using NucleoSpin RNA Kit (Macherey-Nagel) according to the manufacturer's instructions. All samples were examined for quality assessment using Nanodrop One (Thermo Fisher Scientific) and Bioanalyzer (Agilent, Santa Clara, CA, USA). Only samples with an RNA integrity number (RIN) of 7 and higher were used for sequencing library preparation. Libraries for 3′ RNA-seq were prepared using the 3′ method by Lexogen (2) as used in the NGS Competence Center Tübingen (NCCT) where both library preparation and sequencing in randomized batches was performed. First strand synthesis of polyA-tailed RNA from total RNA using oligo dT primers was followed by degradation of the RNA template, second strand synthesis with random primers containing 5′ Illumina-compatible linker sequences and amplification using random primers that add barcodes and cluster generation sequences (2). The libraries were sequenced on the NCCT Nova sequencing platform at a depth of about 10 million reads with 100 bp in length.

Read preprocessing was performed with the Lexogen pipeline (3) that uses *bbduk* of the *BBTools* suite (https://sourceforge.net/projects/bbmap/) for quality trimming. Then,

cleaned reads were aligned with *STAR* (4) to the reference genome GRCh38.104. Differential gene expression analysis was determined with *DESeq2* (5) on genes with at least 50 normalized read counts, and significance thresholds were set at Benjamini-Hochberg adjusted $p_{FDR}<0.05$ and $|\log_2$ fold-change$|>0.5$. Enrichment of affected biological pathways and possible upstream regulators was determined in *Ingenuity Pathway Analysis* (July 2021 version).

Statistical analysis

The data shown were represented by the descriptive statistical quantities of arithmetic mean and standard deviation (SD) of the mean. Statistical analysis was performed using GraphPad Prism 9 software (v9.3.1, GraphPad Software Inc., San Diego, CA, USA) and the test used for normal distribution was the Shapiro-Wilk test. An unpaired Student's *t* test was used for comparisons between two groups, whereas one-way analysis of variance (ANOVA) was used for comparisons among multiple groups. Differences were considered significant if *$p<0.05$. Unless otherwise indicated, the number of independent experiments was n=3, performed in technical duplicates or triplicates. Data from primary cells and our CRISPR/Cas9 *t(4;11)* cells were generated from at least three independent donors.

Supplementary Tables

Supplementary Table 1. List of patient data sets included in the analysis of gent2 database (*http://gent2.appex.kr*).

GSE6401	GSE5820	GSE9476
GSE6477	GSE5788	GSE9874
GSE1133	GSE14286	GSE10631
GSE4475	GSE13996	GSE5580
GSE635	E-MEXP-120	GSE6740
GSE1427	E-MEXP-313	GSE1751
GSE12995	E-TABM-117	GSE2779
GSE6477	E-TABM-125	GSE1010
GSE10255	GSE13591	GSE7893
GSE635	GSE15777	GSE5967
GSE3912	GSE14317	GSE7429
GSE1466	GSE11907	GSE1124
GSE4119	GSE6477	GSE1140
GSE6365	GSE1133	GSE5808
GSE11038	GSE11582	GSE7148
GSE2113	GSE8650	GSE12845
GSE4698	GSE9006	GSE14577
GSE5122	GSE6269	E-AFMX-5
GSE6691	GSE7638	GSE13591
GSE9476	GSE3365	GSE15777
GSE10631	GSE6613	GSE14317
GSE13280	GSE1466	
GSE8970	GSE6236	

Supplementary Table 2. RT-qPCR primer sequences.

Name	Sequence (5‘3‘)
18s rRNA F	CGGCTACCACATCCAAGGAA
18s rRNA R	GCTGGAATTACCGCGGCT
hu *SREBF2* F	CGAATTGAAAGACCTGGTCATG
hu *SREBF2* R	TCCTCAGAACGCCAGACTTGT
hu *HMGCR* F	GAATGTCTTGTGATTGGAGTTGGTA
hu *HMGCR* R	CAAAGCAGCACATAATTTCAAGCT
hu *LDLR* F	CACGGTGGAGATAGTGACAATGTC
hu *LDLR* R	TTTCCTCTGCCAGCAACGT
hu *HMGCS1* F	ACTTGTGCATTCAAACATAGCAACT
hu *HMGCS1* R	GCAGGGAGTCTTGGTACTTTCTTG
hu *SQLE* F	GCCAGCAAGCTTCCTTCCT
hu *SQLE* R	GCGTCTCCCAAAAGAAGAACA
hu *MSMO1* F	TCATCATGAGTTTCAGGCTCCA
hu *MSMO1* R	GGATGTGCATATTCAGCTTCCAT
hu *FDFT1* F	GCCAGGTGCTGGAGGACTT

hu *FDFT1* R	GTATTTCTCAGCCAGATTTCTAAACTC
hu *SC5D* F	CATGATGACCTAGGAGAGTTTCCA
hu *SC5D* R	CAGTGAAAAAGAGGAAAGATATTATACTAACG
hu *ABCA1* F	CTCCCGGAGTTGTTGGAAAC
hu *ABCA1* R	CCTCCGAGCATCTGAGAACAG

Supplementary Table 3. Statistical analyses corresponding to Figure 1B (*SREBF2* expression in healthy and leukemic patient samples [GSE13159, data obtained from *https://servers.binf.ku.dk/bloodspot/*] (6)). Student's *t* test. *p<0.05.

	ALL *t(12;21)*	ALL *t(1;19)*	AML normal karyotype	ALL hyperdyploid	**Pro-B-ALL *t(11q23)/MLL***	CLL	CML	C-/Pre-B-ALL *t(9;22)*	T-ALL	MDS	AML complex.	C-/Pre-B-ALL no *t(9;22)*	AML *inv(16)*	Healthy bone marrow	AML *t(15;17)*	B-ALL *t(8;14)*	AML *t(8;21)*	**AML *KMT2A/MLL***
ALL *t(12;21)*		*	*	ns	*****	*	*	*	*	*	*	*	*	*	*	*	ns	*****
ALL *t(1;19)*	*		ns	ns	**ns**	ns	ns	ns	*	*	*	ns	*	*	ns	ns	ns	*****
AML normal karyotype	*	ns		*	**ns**	*	ns	*	*	*	ns	*	ns	*	ns	ns	*	*****
ALL hyperdyploid	ns	ns	*		*****	*	*	ns	*	ns	*	*	*	ns	ns	*	ns	*****
**Pro-B-ALL *t(11q23)/MLL***	*****	**ns**	**ns**	*****		**ns**	**ns**	**ns**	*****	*****	*****	**ns**	*****	*****	**ns**	**ns**	**ns**	*****
CLL	*	ns	*	*	**ns**		ns	ns	*	*	*	ns	*	*	ns	ns	*	*****
CML	*	ns	ns	*	**ns**	ns		*	*	*	ns	ns	ns	*	ns	ns	*	
C-/Pre-B-ALL *t(9;22)*	*	ns	*	ns	**ns**	ns	*		*	*	*	ns	*	ns	ns	ns	ns	*****
T-ALL	*	*	*	*	*****	*	*	*		*	*	*	ns	*	*	*	*	**ns**
MDS	*	*	*	ns	*****	*	*	*	*		*	*	*	ns	*	*	ns	*****
AML complex.	*	*	ns	*	*****	*	ns	*	*	*		*	ns	*	*	ns	*	**ns**
C-/Pre-B-ALL no *t(9;22)*	*	ns	*	*	**ns**	ns	ns	ns	*	*	*		*	*	ns	ns	*	*****
AML *inv(16)*	*	*	ns	*	*****	*	ns	*	ns	*	ns	*		*	*	ns	*	**ns**
Healthy bone marrow	*	*	*	ns	*****	*	*	ns	*	ns	*	*	*		*	*	ns	*****
AML *t(15;17)*	*	ns	ns	ns	**ns**	ns	ns	ns	*	*	*	ns	*	*		ns	ns	*****
B-ALL *t(8;14)*	*	ns	ns	*	**ns**	ns	ns	ns	*	*	ns	ns	ns	*	ns		ns	**ns**
AML *t(8;21)*	ns	ns	*	ns	**ns**	*	*	ns	*	ns	*	*	*	ns	ns	ns		*****
**AML *KMT2A/MLL***	*****	*****	*****	*****	*****	*****	**ns**	*****	**ns**	*****	**ns**	*****	**ns**	*****	*****	**ns**	*****	

Supplementary Table 4. Statistical analyses corresponding to Supp. Figure 1C (*RORC* expression in healthy and leukemic patient samples [GSE13159, data obtained from *https://servers.binf.ku.dk/bloodspot/*] (6)). Student's *t* test. **p*<0.05.

	ALL *t(12;21)*	ALL *t(1;19)*	AML normal karyotype	ALL hyperdyploid	Pro-B-ALL *t(11q23)/MLL*	CLL	CML	C-/Pre-B-ALL *t(9;22)*	T-ALL	MDS	AML complex.	C-/Pre-B-ALL no *t(9;22)*	AML *inv(16)*	Healthy bone marrow	AML *t(15;17)*	B-ALL *t(8;14)*	AML *t(8;21)*	AML *KMT2A/MLL*
ALL *t(12;21)*		ns	*	*	*	*	ns	*	*	*	*	*	ns	ns	*	*	*	*
ALL *t(1;19)*	ns		*	ns	ns	*	*	*	ns	ns	ns	ns	ns	ns	ns	*	*	*
AML normal karyotype	*	*		ns	*	ns	ns	ns	ns	*	ns	*	ns	*	ns	ns	ns	*
ALL hyperdyploid	*	ns	ns		ns	*	*	ns	ns	ns	ns	ns	ns	ns	ns	ns	*	*
Pro-B-ALL *t(11q23)/MLL*	ns	ns	*	ns		*	*	*	*	ns	ns	ns	ns	ns	ns	*	*	*
CLL	*	*	ns	*	*		ns	ns	ns	*	*	*	ns	*	ns	ns	ns	ns
CML	*	*	ns	*	*	ns		ns	ns	*	*	*	ns	*	ns	ns	ns	ns
C-/Pre-B-ALL *t(9;22)*	*	*	ns	ns	*	ns	ns		ns	*	ns	*	ns	ns	ns	ns	ns	ns
T-ALL	*	ns	ns	ns	*	ns	ns	ns		*	ns	*	ns	ns	ns	ns	ns	ns
MDS	*	ns	*	ns	ns	*	*	*	*		ns	ns	ns	ns	ns	*	*	*
AML complex.	*	ns	ns	ns	ns	*	*	ns	ns	ns		ns	ns	ns	ns	ns	*	*
C-/Pre-B-ALL no *t(9;22)*	*	ns	*	ns	ns	*	*	*	*	ns	ns		ns	ns	ns	*	*	*
AML *inv(16)*	ns	ns	ns	ns	ns	ns	ns	ns	ns	ns	ns	ns		ns	ns	ns	ns	*
Healthy bone marrow	ns	ns	*	ns	ns	*	*	ns	ns	ns	ns	ns	ns		ns	*	*	*
AML *t(15;17)*	*	ns	ns	ns	ns	ns	ns	ns	ns	ns	ns	ns	ns	ns		ns	ns	ns
B-ALL *t(8;14)*	*	*	ns	ns	*	ns	ns	ns	ns	*	ns	ns	ns	*	ns		ns	ns
AML *t(8;21)*	*	*	ns	*	*	ns	ns	ns	ns	*	*	*	ns	*	ns	ns		ns
AML *KMT2A/MLL*	*	*	*	*	*	ns	ns	ns	ns	*	*	*	*	*	ns	ns	ns	

Supplementary Table 5. Top downregulated most differentially expressed genes (DEGs) of *t(4;11)* cells treated with XY018 compared to DMSO control (ranked to padj, genes related to cholesterol metabolism are written in bold).

log2FoldChange	pvalue	padj	Gene
-1,39798318	4,6588E-31	1,3495E-27	***LDLR***
-2,05193556	1,2451E-29	2,7051E-26	***MSMO1***
-1,75211645	3,6002E-29	6,2571E-26	***SQLE***
-1,46674822	4,2897E-26	6,2129E-23	***TMEM97***
-1,5583479	1,0874E-23	1,35E-20	***FDFT1***
-1,86429591	1,8723E-22	2,0338E-19	***HMGCS1***
-2,06090219	2,606E-22	2,5162E-19	*S100A8*
-1,20751037	8,2093E-22	7,1339E-19	*FADS1*
-1,74153799	1,8133E-19	1,3131E-16	***HMGCR***
-1,28663364	5,4494E-19	3,6427E-16	***SC5D***
-0,90347558	9,3291E-17	5,4046E-14	***SREBF2***
-2,44405671	6,4579E-16	3,5075E-13	***DHCR7***
-1,97176976	1,284E-15	6,5637E-13	***DHCR24***
-1,42200338	1,6756E-15	8,0893E-13	***MVK***
-1,84462906	5,012E-14	2,1777E-11	*PNPLA3*
-0,92476183	1,4276E-12	5,9074E-10	***SCD***
-0,950253	2,3924E-11	9,4501E-09	***EBP***
-1,66355796	1,6312E-10	5,6699E-08	***STARD4***
-1,35743249	1,5106E-09	2,9835E-07	*TGFBI*
-1,27329827	1,562E-09	3,0163E-07	*FADS2*
-0,82879812	1,7598E-09	3,3245E-07	*KCNE3*
-1,19879354	2,5328E-09	4,683E-07	*SLC25A1*
-0,69450329	5,9628E-09	1,0575E-06	***AACS***
-1,96341157	8,8532E-09	1,5085E-06	*NFE2*
-1,80482334	1,2048E-07	1,9036E-05	*F13A1*
-0,7931399	1,7237E-07	2,6279E-05	*PLAC8*
-1,59331917	1,9734E-07	2,9567E-05	*LRG1*
-0,83919034	2,8223E-07	4,1569E-05	*NLRC4*
-0,96318157	3,8031E-07	5,4179E-05	*MMAB*
-0,88095377	4,2589E-07	5,9693E-05	*TUBA1A*
-0,47379277	1,2395E-06	0,0001561	*PPDPF*
-1,18993573	1,7311E-06	0,00021188	***INSIG1***
-2,03950448	1,8232E-06	0,00022005	*S100A9*
-1,16204274	2,3817E-06	0,00028352	***MVD***
-0,47944467	2,7106E-06	0,00030993	*ACTG1*
-0,55222706	3,3288E-06	0,00036616	*NCF4*
-0,91268133	3,6508E-06	0,00039656	*ATF5*
-0,67369716	3,7169E-06	0,00039763	*S100A6*
-0,48965061	4,0326E-06	0,00041718	*ITGB2*
-0,58011046	4,685E-06	0,00046796	*RHOU*

-0,47006398	5,7401E-06	0,00056683	*RNASET2*
-0,44420597	5,9116E-06	0,00057721	*ANP32B*
-0,9256395	6,3872E-06	0,00060994	*PRKAR2B*
-1,44666071	6,9435E-06	0,0006488	*SERPINF1*
-0,52455652	6,9173E-06	0,0006488	*H4C3*
-0,75876605	7,0752E-06	0,00065408	*MIR223HG*
-0,4820088	7,7408E-06	0,00068641	*GPI*
-0,42442134	8,1877E-06	0,0007187	*PGD*
-0,73689431	8,3177E-06	0,00072281	*EPB41L4A-AS1*
-0,62211004	8,494E-06	0,00073082	*MXD1*
-0,793365077	1,45502E-05	0,001227581	*C1orf162*
-0,96268822	1,69448E-05	0,001389155	*ARRDC3*
-0,414918232	1,69068E-05	0,001389155	*PGK1*
-1,124896562	1,71714E-05	0,001394574	*RPH3AL-AS1*
-0,618388526	1,83444E-05	0,001474611	*TNFSF13B*
-0,489899328	1,84963E-05	0,001474611	*NXPE3*
-0,462406004	1,97207E-05	0,001557935	*FOSL2*
-1,018729307	2,12641E-05	0,001664732	*CCR2*
-0,438675963	2,28405E-05	0,001772175	*IFITM2*
-0,518436246	2,329E-05	0,001791059	*FCGR2B*
-0,724302412	2,60277E-05	0,001984038	*SIGLEC14*
-1,131643966	3,338E-05	0,00250062	***ERG28***
-0,422926118	3,67371E-05	0,002705471	*APLP2*
-0,818912996	3,88463E-05	0,002836761	***NSDHL***
-1,79523157	4,87517E-05	0,003362316	*BPI*
-1,173908309	4,9991E-05	0,003393919	*PLB1*
-0,877158433	5,47186E-05	0,003576247	*FCGR2C*
-0,822778131	5,40149E-05	0,003576247	*SULT1A1*
-0,757391802	5,42288E-05	0,003576247	*QPRT*
-0,755358735	5,31698E-05	0,003576247	*GBE1*
-0,475748218	5,56259E-05	0,003607378	*TOMM7*
-0,491279499	6,05905E-05	0,003871554	*OSCAR*
-1,575050414	6,233E-05	0,003953631	***IDI1***
-0,527480159	6,84539E-05	0,004256196	*TKT*
-0,40418795	6,90591E-05	0,004256196	*OIP5-AS1*
-0,386074837	6,90547E-05	0,004256196	*PGAM1*
-0,976629126	7,26158E-05	0,004412806	*MLXIPL*
-0,560976996	7,97027E-05	0,004776662	*LDHA*
-0,728816854	8,10051E-05	0,00482147	*CSF3R*
-0,384222814	8,17959E-05	0,004835416	*SEC11A*
-0,476961156	0,000100347	0,005852431	*IRF2BPL*
-0,826530535	0,000112452	0,006471553	*CYTOR*
-0,571972292	0,000117277	0,006704839	*ROGDI*
-0,943068168	0,000118798	0,006747428	*NDRG1*
-0,493524511	0,000123355	0,006915816	*NUDT16L1*
-0,553944386	0,00013357	0,007346354	*MTHFR*
-0,641025003	0,000136634	0,007467586	

-0,446712943	0,000138646	0,007530191	*CKS1B*
-0,884878565	0,000166814	0,008732622	***HSD17B7***
-0,716045839	0,000180235	0,009267714	*SLC46A3*
-0,763450473	0,000182253	0,00931634	*AIF1*

Supplementary Table 6. Top upregulated DEGs of *t(4;11)* cells treated with XY018 compared to DMSO control (ranked to padj, genes related to cholesterol metabolism are written in bold).

log2FoldChange	pvalue	padj	Gene
3,30006868	2,8072E-53	2,4394E-49	***ABCA1***
4,06725785	1,281E-41	5,5661E-38	***ABCG1***
1,29150457	9,1069E-20	7,1945E-17	*MTCO1P40*
1,0108047	4,1568E-18	2,5802E-15	*MTCO1P12*
1,06043552	8,9642E-15	4,1E-12	*MT-TS1*
0,95208639	6,1209E-11	2,3126E-08	*MT-TN*
0,72032363	6,4643E-11	2,3406E-08	*CERT1*
0,80813702	3,4736E-10	9,1449E-08	*NEAT1*
0,8393719	3,9066E-10	9,1449E-08	***RNF145***
1,21572517	4,1271E-10	9,1449E-08	*RNA5S15*
1,21572517	4,1271E-10	9,1449E-08	*RNA5S16*
1,21646942	4,5251E-10	9,1449E-08	*RNA5S1*
1,21646942	4,5251E-10	9,1449E-08	*RNA5S2*
1,21646942	4,5251E-10	9,1449E-08	*RNA5S3*
1,21646942	4,5251E-10	9,1449E-08	*RNA5S4*
1,21646942	4,5251E-10	9,1449E-08	*RNA5S5*
1,21646942	4,5251E-10	9,1449E-08	*RNA5S6*
1,21646942	4,5251E-10	9,1449E-08	*RNA5S7*
1,21646942	4,5251E-10	9,1449E-08	*RNA5S8*
1,21646942	4,5251E-10	9,1449E-08	*RNA5S10*
1,21646942	4,5251E-10	9,1449E-08	*RNA5S11*
1,21646942	4,5251E-10	9,1449E-08	*RNA5S12*
1,21646942	4,5251E-10	9,1449E-08	*RNA5S13*
1,21646942	4,5251E-10	9,1449E-08	*RNA5S14*
1,21646942	4,5251E-10	9,1449E-08	*RNA5S17*
0,81066124	2,6556E-09	4,8077E-07	*MYLIP*
1,22933232	7,2657E-09	1,2628E-06	*SELL*
0,53964992	1,3445E-08	2,2469E-06	*MT-RNR2*
0,71189136	3,4257E-08	5,6169E-06	*MTRNR2L3*
0,63072599	4,2151E-08	6,7832E-06	*ASAH1*
0,86496297	1,36E-07	2,1105E-05	*STS*
0,76161511	3,7661E-07	5,4179E-05	*LPCAT1*
1,31660903	4,4429E-07	6,1283E-05	*TNS1*
0,89599323	4,7169E-07	6,4047E-05	*LGALS3*

0,78045386	6,4065E-07	8,565E-05	*BTD*
0,63753322	7,3714E-07	9,7057E-05	*MT-CO1*
1,40769834	1,0893E-06	0,00014129	*MTSS1*
1,86662953	1,1258E-06	0,00014388	*TFRC*
0,751778	1,3913E-06	0,00017272	*WASF1*
1,25233252	2,578E-06	0,00030274	*PPP1R27*
0,55096739	2,6488E-06	0,00030691	*FNIP1*
0,57712323	2,9904E-06	0,00033748	*CALU*
0,7510253	3,1456E-06	0,00035045	*MTRNR2L6*
0,47155898	3,7978E-06	0,00039763	*MBNL1*
0,6374291	3,7933E-06	0,00039763	*MTCO2P12*
0,62351577	4,1386E-06	0,00042311	*PAN3*
1,03114317	4,2599E-06	0,00043044	*ANKRD28*
0,62871492	6,2548E-06	0,00060393	*PLIN2*
0,55020699	7,4048E-06	0,00067651	*CAPG*
0,62401754	7,4736E-06	0,00067651	*MTND6P3*
0,846842188	7,56878E-06	0,000678069	*MT-TA*
0,59976656	1,36119E-05	0,001159681	*MT-CO2*
0,599036062	1,56736E-05	0,001309653	*BIVM*
0,690712647	2,63293E-05	0,001989576	*MT-ATP6*
0,552166641	3,45145E-05	0,002563514	*STX3*
0,71712826	4,06946E-05	0,002946969	*PDGFC*
0,424042485	4,12856E-05	0,00296506	*ARL8B*
0,683881766	4,53325E-05	0,00322901	*HIVEP1*
0,650294432	4,65663E-05	0,003289928	*RASAL2*
0,460905904	4,7024E-05	0,00329547	*GOLGA4*
0,795066295	4,80608E-05	0,003341185	*CAMK1D*
0,451649367	4,95394E-05	0,003389745	*SLC39A7*
0,45801416	5,47343E-05	0,003576247	*NFE2L2*
1,161504218	5,92441E-05	0,003813566	*ENPP2*
0,79346386	6,46046E-05	0,004068218	*CAMSAP2*
0,655460926	6,96471E-05	0,004262209	*CREB3*
0,416373308	7,56131E-05	0,004563038	*NFATC1*
0,493968222	9,83416E-05	0,00577425	*MT-CYB*
0,796859354	0,000104879	0,006076002	*SCFD2*
0,894045185	0,000123163	0,006915816	*RELB*
0,751611775	0,000126436	0,007043137	*LTB*
0,871842151	0,000128625	0,007119415	*PITPNC1*
0,542707442	0,00014164	0,007645066	*CYFIP1*
0,454989576	0,000147423	0,007908063	*TUT4*
0,428542553	0,000148585	0,007921486	*MTRNR2L12*
0,433367252	0,000162	0,00858403	*MTRNR2L8*
0,507853617	0,000165467	0,008714601	*MT-ATP8*
0,70112291	0,000173349	0,009020354	*PDGFA*
0,77727253	0,000177238	0,009167847	*MEMO1*
0,402211614	0,000183497	0,009325064	*MT-RNR1*
0,647116533	0,000191639	0,009636629	*MGLL*

0,411439242	0,00024883	0,012356178	*FLT3*
0,3973053	0,000322054	0,015722739	*CELF2*
0,551264808	0,000324142	0,015736278	*MTATP8P1*
0,643229014	0,000340186	0,016332692	*MT-TE*
0,414123891	0,000347673	0,016600442	*GLS*
0,472233097	0,000375523	0,017587591	*LILRB1*
0,560446115	0,000428578	0,019810335	*MTMR10*
0,462685044	0,000476325	0,021446979	*TTC3P1*
0,930307405	0,000485154	0,021731889	*UBASH3B*
0,728938509	0,000501737	0,022245377	*ENAH*
0,811988275	0,000513991	0,022673002	*RHOBTB1*
0,728026224	0,000520637	0,02273535	*LINC00641*
0,310211963	0,000555654	0,024143169	*WDR43*
0,375114513	0,000572553	0,024631105	*LCORL*
0,975532998	0,000584851	0,025036251	*IGSF6*
0,437282794	0,000590116	0,025137805	*RALGDS*
0,349147973	0,000679143	0,028373817	*ALG5*

Supplementary Figures

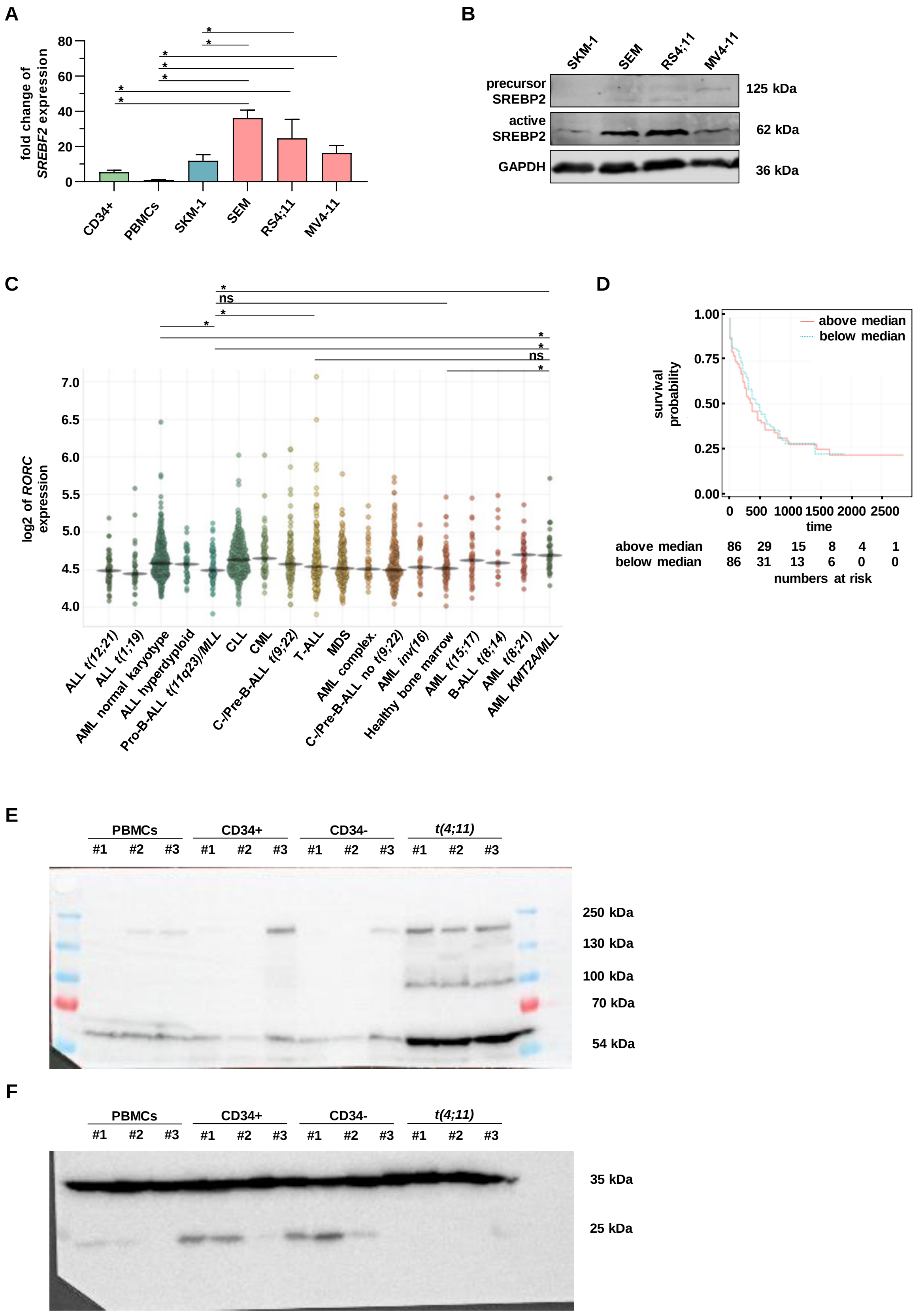

Supp. Figure 1. ***t(4;11)*** **cell lines express high levels of SREBP2 and** ***RORC***

expression is enhanced in *KMT2A*r leukemia patients. (A) *SREBF2* mRNA expression was analyzed with RT-qPCR. huCB-derived CD34+ HSPCs and PBMCs from healthy donors were compared to SKM-1 and *t(4;11)* cell lines whereas PBMCs were set as 1. Data are shown as mean ± SD. n=3. One-way ANOVA. *p<0.05. **(B)** Representative western blot analysis of full length and N-terminal SREBP2 in SKM-1 and *t(4;11)* cell lines. GAPDH was used as loading control. **(C)** *RORC* expression in healthy and leukemic patient samples (GSE13159, data obtained from *https://servers.binf.ku.dk/bloodspot/*). The corresponding statistical analyses are shown in Supp. Table 4. **(D)** Kaplan-Meier survival curves from leukemic patients with $RORC^{low}$ (blue line) and $RORC^{high}$ (red line) according to the median value of *RORC* using data from TCGA (obtained from *https://servers.binf.ku.dk/bloodspot/*). Student's *t* test. *p<0.05. Uncropped data figure including molecular weight marker for images shown in Figure 1F. Representative Western blot analysis of full length and N-terminal SREBP2 **(E)** and GAPDH as loading control **(F)** in PBMCs from healthy donors, CD34+ HSPCs and CD34- cells from huCB and CRISPR/Cas9 *t(4;11)* cells.

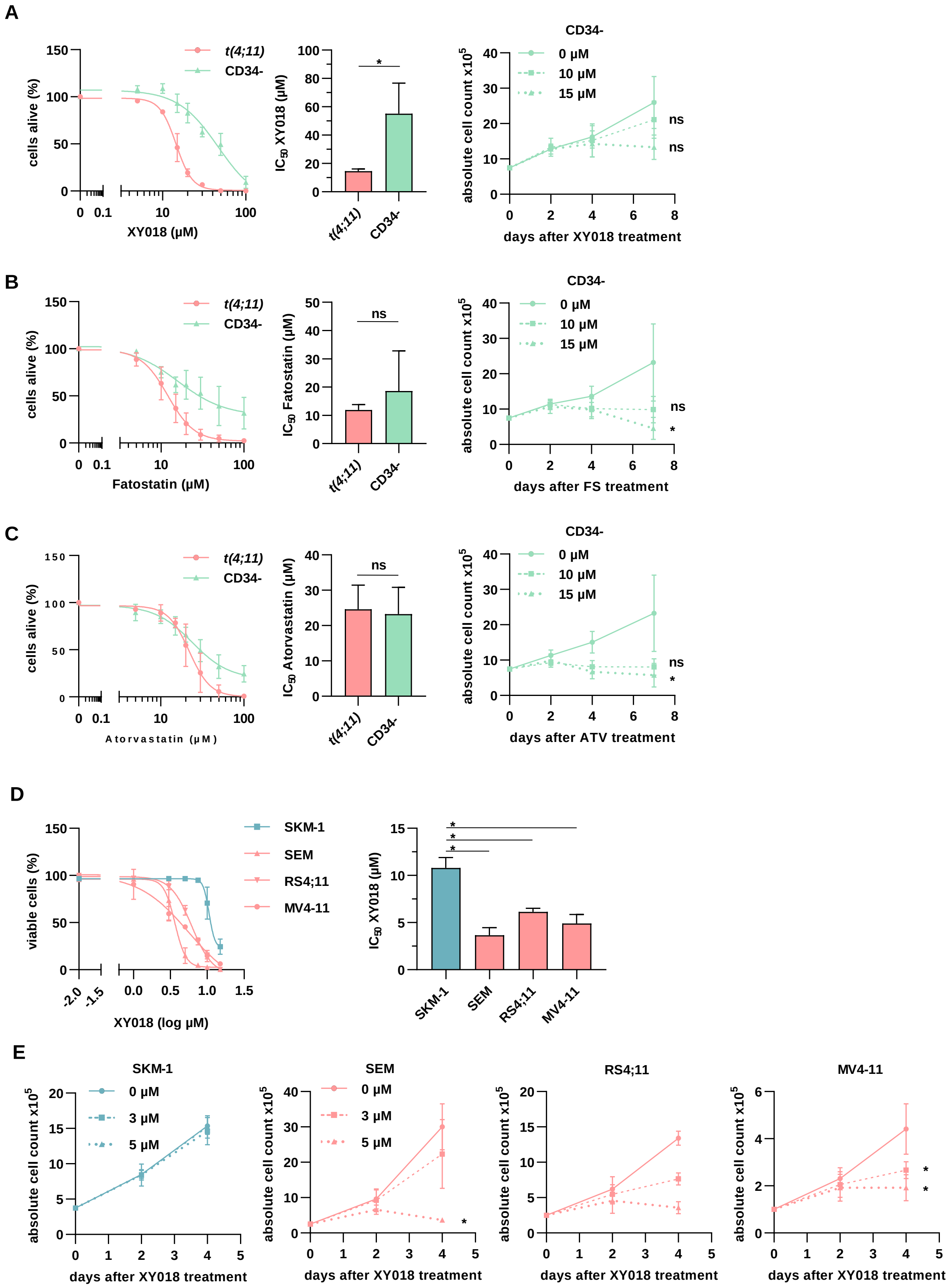
A
cells alive (%)
t(4;11)
CD34-
XY018 (µM)
IC50 XY018 (µM)
*
CD34-
absolute cell count x10^5
0 µM
10 µM
15 µM
ns
ns
days after XY018 treatment
B
cells alive (%)
t(4;11)
CD34-
Fatostatin (µM)
IC50 Fatostatin (µM)
ns
CD34-
absolute cell count x10^5
0 µM
10 µM
15 µM
ns
*
days after FS treatment
C
cells alive (%)
t(4;11)
CD34-
Atorvastatin (µM)
IC50 Atorvastatin (µM)
ns
CD34-
absolute cell count x10^5
0 µM
10 µM
15 µM
ns
*
days after ATV treatment
D
viable cells (%)
SKM-1
SEM
RS4;11
MV4-11
XY018 (log µM)
IC50 XY018 (µM)
*
*
*
E
SKM-1
SEM
RS4;11
MV4-11
absolute cell count x10^5
0 µM
3 µM
5 µM
*
*
*
days after XY018 treatment

F

G

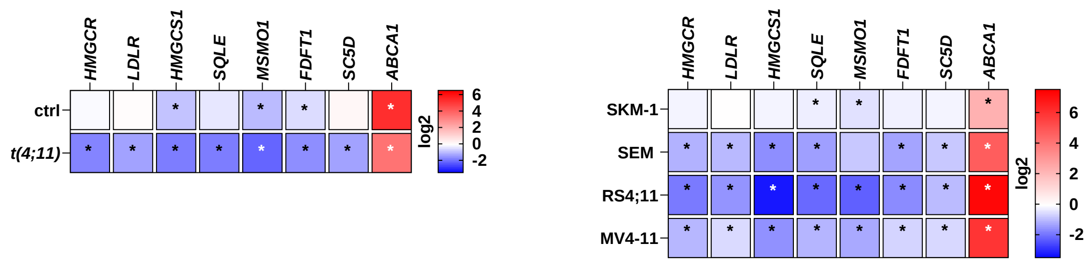

Supp. Figure 2. Cells harboring *t(4;11)* are more sensitive to the RORγ antagonist than control cells. CD34- cells were treated with DMSO (0 µM) or increasing concentrations of XY018 **(A)**, fatostatin **(B)** or atorvastatin **(C)** for seven days. The percentage of living cells (annexin V-, PI-) was evaluated with flow cytometry and IC_{50} values were interpolated from a four-parameter logistic model constrained to 0 and 1 in GrapdhPad Prism. Cells were counted with trypanblue and absolute cell count was evaluated for different inhibitor concentrations as indicated. Experiments were performed with three independent donors (n=3) in technical triplicates and dots represent the mean ± SD. One-way ANOVA. *p<0.05. ns=not significant. **(D)** Cell lines were treated with increasing concentrations of XY018 for four days. The percentage of living cells (annexin V-, PI-) was evaluated with flow cytometry. IC_{50} values were interpolated from a four-parameter logistic model constrained to 0 and 1 in GrapdhPad Prism. Data are shown as mean ± SD. n=3. One-way ANOVA. *p<0.05. **(E)** Cells were counted with trypanblue and absolute cell count was evaluated for different inhibitor concentrations (0 µM, 3 µM, 5 µM) of XY018. Data are shown as mean ± SD. n=3. One-way ANOVA. *p<0.05. **(F)** Heat map display of fold changes (in log2) of cholesterol pathway-related target gene expression after XY018 treatment in *t(4;11)* and CD34+ HSPCs (ctrl). Cells were treated with 15 µM XY018 or DMSO for seven days and analyzed with RT-qPCR. Each square represents the mean of three independent donors (n=3) ± SD. Student's *t* test. *p<0.05. **(G)** Heat map display of fold

changes (in log2) of cholesterol pathway-related target gene mRNA after XY018 treatment. Cell lines were treated with 3 µM XY018 or DMSO for four days and analyzed with RT-qPCR. Each square represents the mean ± SD. n=3. Student's *t* test. **p*<0.05.

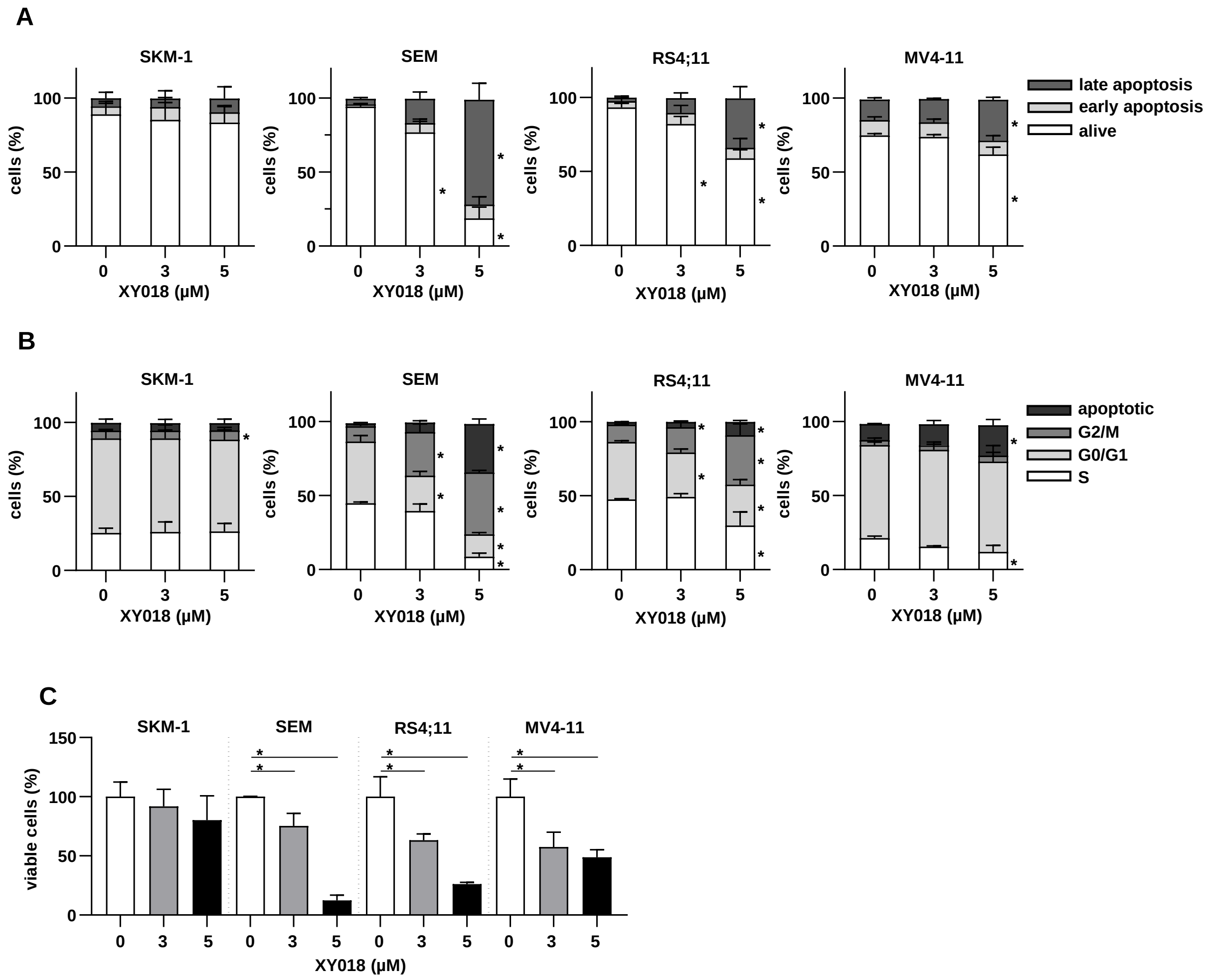

Supp. Figure 3. Inhibition of RORγ induces apoptosis and changes cell cycle in *t(4;11)* cell lines. (A) SKM-1 and *t(4;11)* cell lines were treated with indicated concentrations of XY018 for four days. Histograms show the percentage of cells in different apoptotic stages as mean ± SD. n=3. One-way ANOVA. **p*<0.05. **(B)** Cells were treated as described in (A) and analyzed for cell cycle using flow cytometry. **(C)**

For analysis of cell viability, cells were measured using alamarBlue assay. n=3. One-way ANOVA. *$p<0.05$.

A

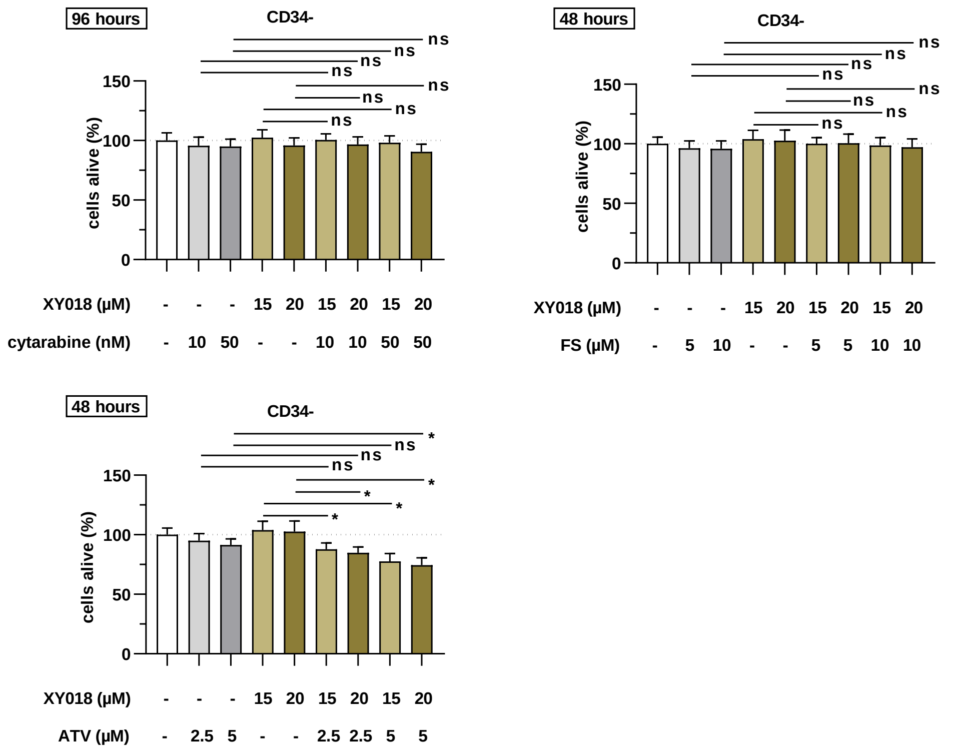

B

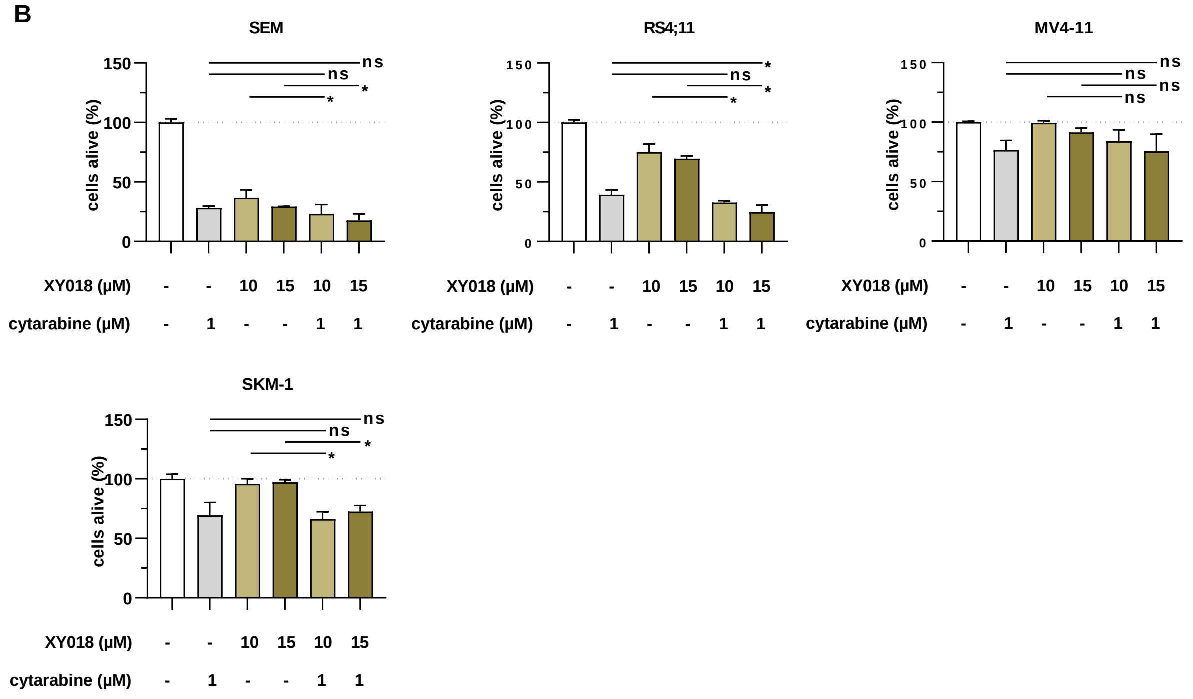

C

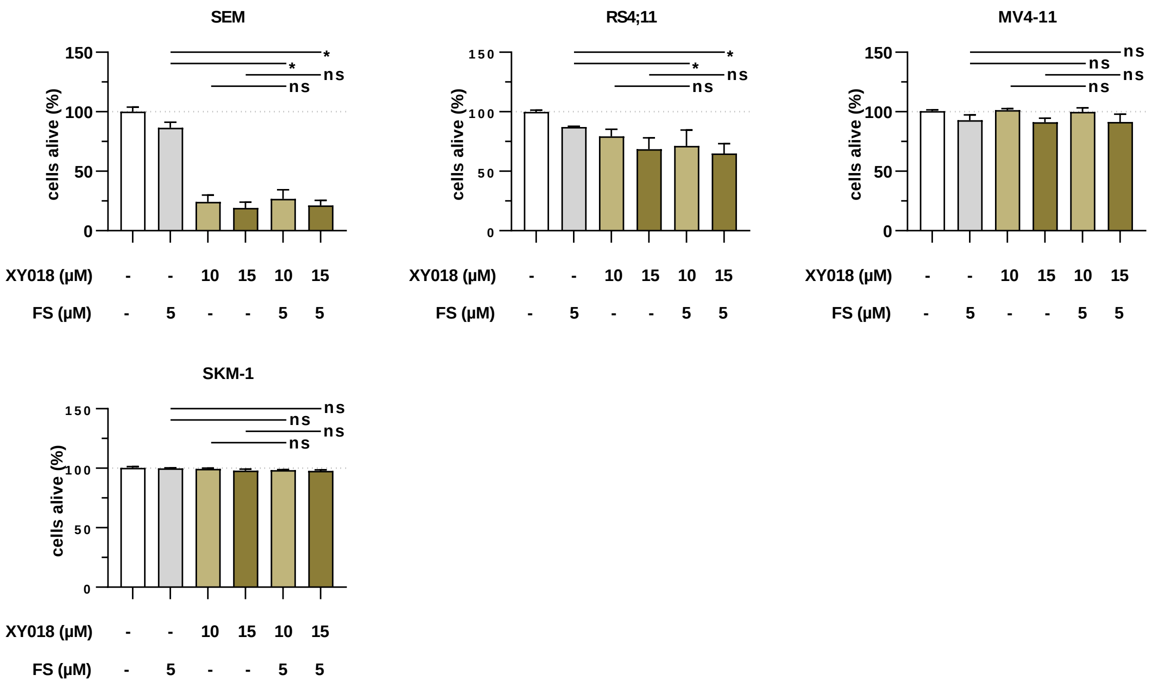

D

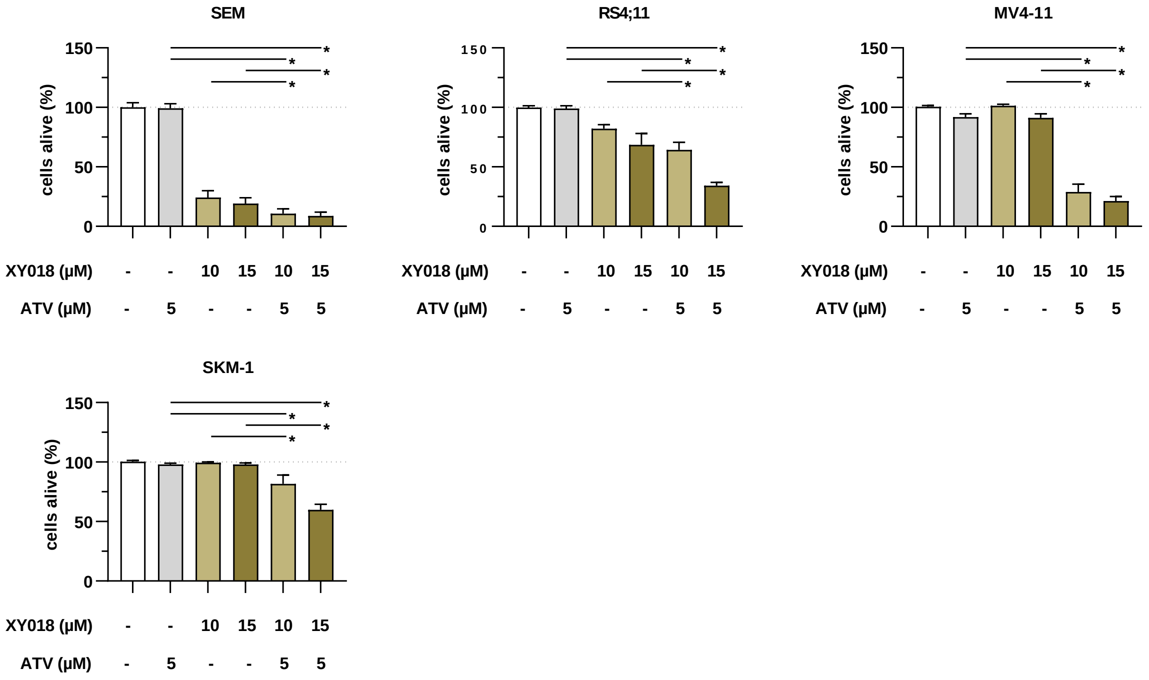

Supp. Figure 4. Inhibition of RORγ in combination with cytarabine and cholesterol-modulating agents in CD34- cells and cell lines. (A) CD34- control cells were treated with XY018 alone or in combination with various concentrations of cytarabine, FS, or ATV as indicated. The percentage of living cells (annexin V-, PI-)

was evaluated with flow cytometry, and vehicle-treated cells were set to 100 as an internal control. Bars represent the mean of three independent donors (n=3) ± SD. One-way ANOVA. *$p<0.05$. ns=not significant. *t(4;11)* cell lines (SEM, RS4;11 and MV4-11) and SKM-1 cells were treated with 10 µM or 15 µM XY018 alone or in combination with 1 µM cytarabine **(B)**, 5 µM FS **(C)**, or 5 µM ATV **(D)** for 48 hours. Bars represent the mean of three independent experiments (n=3) ± SD. One-way ANOVA. *$p<0.05$. ns=not significant.

1. Secker KA, Bloechl B, Keppeler H, Duerr-Stoerzer S, Schmid H, Schneidawind D, et al. MAT2A as Key Regulator and Therapeutic Target in MLLr Leukemogenesis. Cancers (Basel). 2020;12(5).
2. Ma F, Fuqua BK, Hasin Y, Yukhtman C, Vulpe CD, Lusis AJ, et al. A comparison between whole transcript and 3' RNA sequencing methods using Kapa and Lexogen library preparation methods. BMC Genomics. 2019;20(1):9.
3. Lexogen. QuantSeq 3‘ mRNA-Seq Integrated Data Analysis Pipelines on BlueBee® Genomics Platform 2012 [
4. Dobin A, Davis CA, Schlesinger F, Drenkow J, Zaleski C, Jha S, et al. STAR: ultrafast universal RNA-seq aligner. Bioinformatics. 2013;29(1):15-21.
5. Love MI, Huber W, Anders S. Moderated estimation of fold change and dispersion for RNA-seq data with DESeq2. Genome Biol. 2014;15(12):550.
6. Bagger FO, Sasivarevic D, Sohi SH, Laursen LG, Pundhir S, Sonderby CK, et al. BloodSpot: a database of gene expression profiles and transcriptional programs for healthy and malignant haematopoiesis. Nucleic Acids Res. 2016;44(D1):D917-24.
